# Assessing the information quality of AI-generated patient educational materials for diabetes: a scoping review

**DOI:** 10.3389/fpubh.2026.1902424

**Published:** 2026-08-05

**Authors:** Jingwen Song, Norafisyah Makhdzir, Zarina Haron, Chunping Qin, Li Wang

**Affiliations:** 1 Faculty of Medicine and Health Sciences, Universiti Putra Malaysia, Seri Kembangan, Selangor, Malaysia; 2 Nursing Department, The Fifth Affiliated Hospital of Guangxi Medical University, Nanning, China; 3 Department of Nursing, Faculty of Medicine and Health Sciences, Universiti Putra Malaysia, Seri Kembangan, Selangor, Malaysia; 4 Department of Geriatrics, The Fifth Affiliated Hospital of Guangxi Medical University, Nanning, China

**Keywords:** artificial intelligence, diabetes, information quality, patient education materials, scoping review

## Abstract

**Objective:**

To map the evidence on artificial intelligence (AI)-generated diabetes-related patient education materials and patient-facing health information, with particular attention to AI models, prompting approaches, evaluation methods, and information-quality outcomes.

**Methods:**

This scoping review was conducted in accordance with the JBI methodology for scoping reviews and reported following the PRISMA-ScR checklist. The review was registered on the Open Science Framework (doi: 10.17605/OSF.IO/U4FAE) PubMed, Web of Science, Embase, Scopus, Cochrane CENTRAL, CNKI, WanFang Data, and SinoMed were searched from inception to May 1, 2026. Chinese- and English-language literature was searched. Two reviewers independently screened studies, charted data, and mapped reported outcomes to Wang and Strong's information quality framework. Outcomes not adequately represented by the framework were retained as additional dimensions. Descriptive statistics and narrative synthesis were used.

**Results:**

Of 6,049 records identified, 24 studies from 11 countries or regions were included. All studies evaluated ChatGPT or another GPT-family model; 21 used zero-shot or direct prompting, three used role prompting, and two implemented retrieval-augmented generation. Eleven indicators were mapped to the information quality framework, with ease of understanding (*n* = 14), accuracy (*n* = 13), and believability (*n* = 9) assessed most frequently. Six additional outcomes were identified: clinical safety (*n* = 5), actionability (*n* = 3), response efficiency (*n* = 1), personalization (*n* = 1), transparency (*n* = 1), and empathy (*n* = 1). Most studies reported reading demands above those generally recommended for patient education, although findings varied by language, material type, and assessment method. Study-specific instruments were used in 17 studies (70.8%), whereas 10 (41.7%) used structured or established tools. Only six studies reported full source or model blinding, 10 reported quantitative inter-rater agreement, and three involved patients or members of the public.

**Conclusion:**

Research on AI-generated diabetes education is expanding, but substantial heterogeneity in prompts, evaluators, tools, and outcome definitions limits comparison across studies. Future research should prioritize validated, multilingual, and patient-centered evaluation tools that integrate conventional information-quality attributes with clinically relevant dimensions such as safety, actionability, personalization, transparency, empathy, and response efficiency.

## 1 Introduction

According to the global summary analysis by the Non-Communicable Disease (NCD) Risk Factor Collaboration, an estimated 828 million adults aged 18 years and over worldwide had diabetes in 2022. At the same time, approximately 445 million adults aged 30 years and above with diabetes were untreated, representing 59% of diabetes patients in this age range (1). With systematic analysis of the Global Burden of Diseases (GBD) 2021, it is further predicted that the number of diabetes patients worldwide may exceed 1.31 billion by 2050 (2).

Managing diabetes poses unique challenges in terms of the sheer number of patients, long-term management that relies heavily on patients' daily self-management, complex nutritional guidance, and continuous monitoring. Diabetes Self-Management Education and Support (DSMES) is regarded as a crucial part of diabetes care, providing diabetes patients with the knowledge, skills, and confidence to assume self-management responsibilities (3, 4). High-quality patient education materials are an important medium to connect professional medical advice with patients' daily self-management behaviors. However, the information transfer during traditional medical encounters has limitations. Kessels' review shows that 40%−80% of the information given by health care professionals may be forgotten immediately, and almost half of the information that is remembered is wrong (5). Therefore, there is a need to explore new patient education methods to provide accurate, clear, and understandable health education information to support the long-term self-management of diabetes patients.

Generative artificial intelligence (AI) has evolved rapidly. Research has demonstrated that AI-generated patient education materials (PEMs) are being employed in critical domains, including patient education and discharge instructions (6, 7). However, patient education materials generated by generative AI are not perfect and may contain issues such as inaccurate medical information, hallucinations, and neglect of the unique characteristics of patients with diabetes. Therefore, a systematic evaluation of their quality is necessary before using them to support patient care.

Several recent scoping reviews have synthesized the broader use of large language models in patient education. Aydin reviewed medical applications of large language models in patient education and engagement across multiple clinical contexts (8). The applications identified were generation of patient education materials, interpretation of medical information, lifestyle recommendations, support for medication use, perioperative care instructions, and optimization of doctor-patient interaction. However, this review mainly focuses on the application scenarios of large language models in various medical specialties. It did not integrate theoretical models to comprehensively analyze the information quality of patient education materials for diabetes.

AlSammarraie and Househ also identified studies on the use of large language models for generating patient education materials, including the models used, variables related to prompts, evaluation measures, assessment tools, retrieval-augmented approaches, and comparative results (9). The most commonly assessed indicators were accuracy and readability, while standardized assessment frameworks, patient involvement, and non-English prompts received less attention. But there remain gaps in patient education that is specific to diabetes. The search was limited to English language studies published up to September 2024, and the review did not specifically synthesize AI-generated diabetes-related patient education materials through a theory framework. It remains unclear which indicators of information quality have been assessed in diabetes-focused studies, which tools have been used, and which aspects of information quality have been underexamined. The information quality framework of Wang and Strong is a key theoretical foundation for our scoping review (10). The framework categorizes information quality into four main categories based on the user's perspective of information: intrinsic quality, contextual quality, representational quality, and accessibility quality. These categories include many of the traditional quality attributes, such as accuracy, believability, relevance, completeness, timeliness, understandability, and accessibility. However, the assessment of medical content generated by generative AI needs to be critically considered further, as such content may include failure modes that are less explicitly represented in traditional information quality frameworks. These include hallucinated but seemingly authoritative responses, unclear provenance of evidence, delayed knowledge updating, and variability in outputs across prompts. Against this backdrop, Sallam called for a more comprehensive benchmarking approach for generative AI in healthcare, noting that evaluation should encompass not only accuracy but also broader dimensions such as diversity and consistency (11). Hence, when evaluating AI-generated information for diabetes patient education, it should draw on existing information quality theory as an organizing framework, and additionally consider quality risks specific to generative AI.

This scoping review aims to map the evidence base for AI-generated patient education materials and patient-facing health information on diabetes. We aimed to identify the AI models, prompts or input materials, evaluation tools, and information-quality indicators used in previous studies and classify the reported outcomes based on Wang and Strong's information quality framework.

The research questions of this review are:

Q1. What AI models and input materials have been used to create diabetes patient education materials or patient facing health information?

Q2. What assessment tools and reference standards have been utilized?

Q3. What quality indicators of information were evaluated?

Q4. What are the evidence gaps for the evaluation and reporting of AI-generated patient education materials related to diabetes?

## 2 Methods

### 2.1 Study design and registration

This scoping review was conducted in accordance with the Joanna Briggs Institute (JBI) methodology for scoping reviews. We reported following the Preferred Reporting Items for Systematic Reviews and Meta-Analyses Extension for Scoping Reviews (PRISMA-ScR) (12). The PRISMA-ScR checklist is available in Supplementary Document 1. This study has been registered on the Open Science Framework, registration doi: 10.17605/OSF.IO/U4FAE.

The eligibility criteria were developed using the Population–Concept–Context (PCC) framework. In this review, the PCC framework consisted of the following components.

“Population” referred to adults with diabetes, individuals with diabetes-related complications, or members of the public seeking diabetes-related health education information. Diabetes-related conditions included type 1 diabetes, type 2 diabetes, gestational diabetes mellitus, and diabetes-related complications such as diabetic kidney disease, diabetic retinopathy, and diabetic foot ulcers.

“Concept” referred to AI-generated diabetes-related patient education materials or patient-oriented health information. These materials were operationally defined as text-based health information generated by an artificial intelligence model in response to explicit prompts, questions, or input materials, intended for patients or the public. Eligible AI-generated outputs included educational articles, brochures, guides, discharge instructions, and responses to patient-oriented or public-facing diabetes-related questions. To meet the concept criterion, studies were required to report the AI model used, describe the prompts, questions, or input materials, and evaluate at least one information quality-related indicator, such as accuracy, completeness, believability, readability, understandability, actionability, relevance, appropriateness, safety, clarity, or comprehensiveness.

“Context” referred to diabetes-related patient education, health information seeking, digital health, and AI-assisted health communication settings. No restrictions were applied regarding country, region, healthcare system, or specific care setting. However, the AI-generated content had to be relevant to patient education or public-facing health information rather than clinician-facing diagnosis, treatment decision-making, or examination answering.

The PCC framework directly guided study selection. During title and abstract screening, records were first assessed for relevance to diabetes or diabetes-related complications, AI-generated patient-oriented educational content, and a health education or digital health context. During full-text screening, each study was assessed against all three PCC components and the predefined inclusion and exclusion criteria. Studies that failed to meet one or more PCC components, or in which diabetes-specific patient education outputs could not be distinguished, were excluded.

### 2.2 Search strategy

The following eight databases were searched from inception to May 1, 2026: PubMed, Web of Science, Embase, Scopus, Cochrane Central Register of Controlled Trials (CENTRAL), China National Knowledge Infrastructure (CNKI), WanFang Data, and SinoMed. The search strategy was developed in consultation with a health sciences librarian with expertise in systematic review searching. The researchers used Chinese and English as retrieval languages. The search strategy was designed with conceptual blocks: (1) diabetes-related terms, (2) artificial intelligence/generative AI/chatbot terms, and (3) patient education/information/health communication terms. The complete search strategies for all databases are provided in Supplementary Document 2.

Additionally, we performed backward citation searching by reviewing the reference lists of all included studies and forward citation searching using Web of Science for key included studies.

Database selection was based on the need to cover biomedical and multidisciplinary health literature. IEEE Xplore and ACM Digital Library were not included, as the primary focus was on clinical information quality evaluation rather than technical AI development; their absence is acknowledged as a limitation.

### 2.3 Inclusion and exclusion criteria

Studies were included if they met the following criteria: (1) the population included adults with diabetes, individuals with diabetes-related complications, or members of the public seeking diabetes-related health education information; (2) the study evaluated AI-generated diabetes-related patient education materials or patient-oriented health information, including educational articles, brochures, guides, discharge instructions, or responses to patient-oriented diabetes-related questions; (3) the AI-generated content was produced using clearly described prompts, questions, or input materials; (4) the AI model used was clearly reported; (5) at least one information quality-related indicator was evaluated, such as accuracy, completeness, reliability, readability, understandability, actionability, relevance, appropriateness, safety, clarity, comprehensiveness, or trustworthiness; (6) the evaluation tools, criteria, or scoring methods were reported; (7) the study was an original quantitative study published in a peer-reviewed journal; and (8) the publication language was Chinese or English.

Studies were excluded if they met any of the following criteria: (1) the study did not focus on diabetes or diabetes-related complications; (2) the AI-generated output was not patient-oriented or public-facing health education information; (3) the study mainly focused on clinician-facing diagnosis, treatment decision-making, administrative tasks, prediction modeling, or technical documentation; (4) the study did not evaluate any information quality-related indicators or did not report evaluation tools, criteria, or scoring methods; (5) the publication type was a conference abstract, editorial, commentary, letter, protocol, review, or other non-original study; or (6) the full text was not available through reasonable means.

### 2.4 Study selection and data charting

Two researchers independently extracted data after reading the entire text word by word. We systematically charted the evaluation methods used in all included studies but did not conduct a formal methodological quality or risk-of-bias assessment.

The general characteristics of literature include author, publication year, country, disease/topic, AI output types, AI model, and information-quality indicators.

The structured methodological charting framework includes prompt language, session structure, AI input type frequency, prompting types, evaluators, evaluator blinding, inter-rater agreement, and tool types.

Inter-reviewer agreement was assessed before consensus discussions. Cohen's kappa was calculated separately for title and abstract screening and full-text screening, while percentage agreement was calculated for data extraction. Disagreements were subsequently resolved through discussion or consultation with a third reviewer. The full names and abbreviations of the professional terms are detailed in Supplementary Document 2.

### 2.5 Data items

The variables for which data were sought, together with their operational definitions, assumptions, and simplifications, are provided in Supplementary Document 3.

### 2.6 Data synthesis

In accordance with established methodological guidance for scoping reviews, the extracted data were summarized using descriptive statistics and narrative synthesis (13, 14). Because the included studies differed substantially in their AI models, diabetes-related topics, prompt designs, assessment methods, and outcome reporting, statistical pooling was neither appropriate nor planned.

Frequencies were calculated for disease or topic, AI output types, AI model, prompt language, session structure, AI input types, prompting strategy, evaluators, evaluator blinding, inter-rater agreement, and tool types.

To ensure reproducibility in consolidating heterogeneous evaluation terms, we developed explicit coding rules and a coding matrix to define how original indicators were grouped and mapped to Wang and Strong's information-quality domains. The theoretical framework is provided in Supplementary Document 4, and the coding matrix is provided in Supplementary Document 5. The coding rules specified the coding unit, retention criteria, and criteria for consolidating conceptually equivalent terms.

The coding unit was an independently reported evaluation dimension, scoring item, tool subscale, classification outcome, or computational metric used to assess AI-generated diabetes-related patient education materials or patient-facing health information.

An item was retained only when it met at least one of the following criteria: (1) it was assessed with an explicit scale, tool, formula, classification rule, reference standard or computational method; (2) it was reported in the Results section as a score, proportion, category, or comparison; or (3) it represented a clearly defined subdomain of a validated or study-specific assessment tool. Descriptive statements without an independent assessment method or reported result were not coded.

Conceptually similar terms were consolidated when they referred to the same object of assessment, captured the same evaluative construct, relied on comparable evidence or scoring criteria, and could be merged without changing the meaning intended by the original authors.

Each original indicator was assigned to one primary dimension. If a tool reported separate subscales, each subscale was coded separately; if only a total score was reported, it was coded once according to the dominant construct described in the study. Outcomes that did not fit Wang and Strong's original framework were retained as additional evaluated outcomes rather than forced into the four information-quality domains.

Two reviewers independently performed the consolidation and mapping. Cohen's kappa was calculated separately for indicator retention and primary-dimension mapping. Disagreements were resolved by re-examining the original articles, particularly the descriptions of the assessment tools, scoring criteria, and results tables. A third reviewer was consulted when consensus could not be reached.

## 3 Results

**Figure 1:**
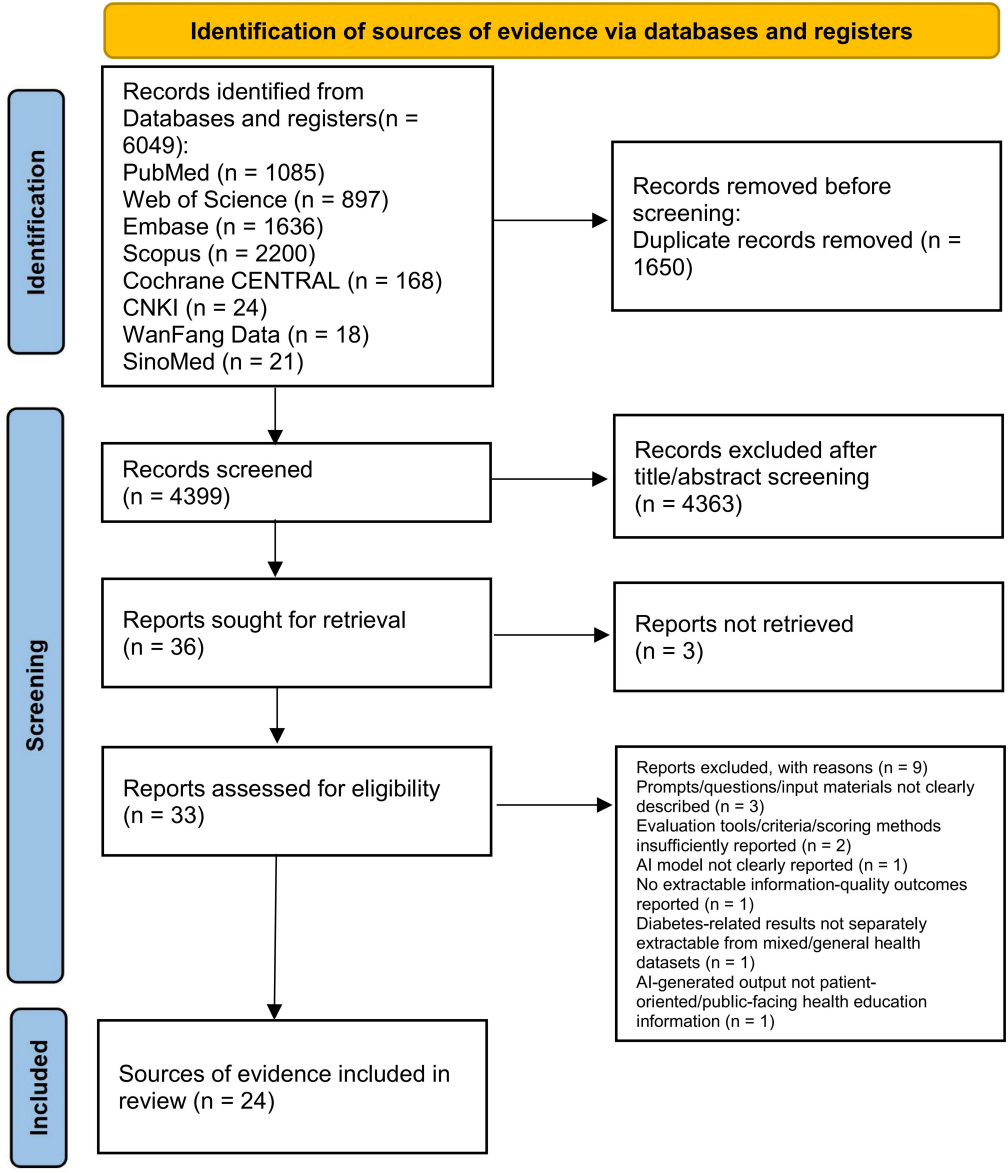
PRISMA-ScR flow diagram of the source selection process.

A total of 6,049 records were initially identified. All search results were imported into EndNote X9, and duplicate records were removed using the software-assisted deduplication function followed by manual checking. After duplicates were excluded, 4,399 records remained for title and abstract screening. Thirty three full-text articles were then independently assessed by two researchers, and disagreements were resolved through discussion or, when necessary, consultation with a third researcher. Finally, 24 studies were included in the review. The study selection process was documented using the PRISMA flow diagram (15), as shown in Figure 1, PRISMA-ScR flow diagram of the source selection process.

Before consensus discussions, the Cohen's kappa values were 0.72 for title and abstract screening and 0.79 for full-text screening. The percentage agreement for data extraction was 90.6%. For the consolidation and mapping process, Cohen's kappa was 0.80 for indicator retention and 0.76 for primary-dimension mapping. All disagreements were subsequently resolved through discussion, with a third reviewer consulted when consensus could not be reached.

### 3.1 General demographics

The general characteristics of the literature are presented in Supplementary Document 6, General characteristics of the 24 included studies.

**Figure 2:**
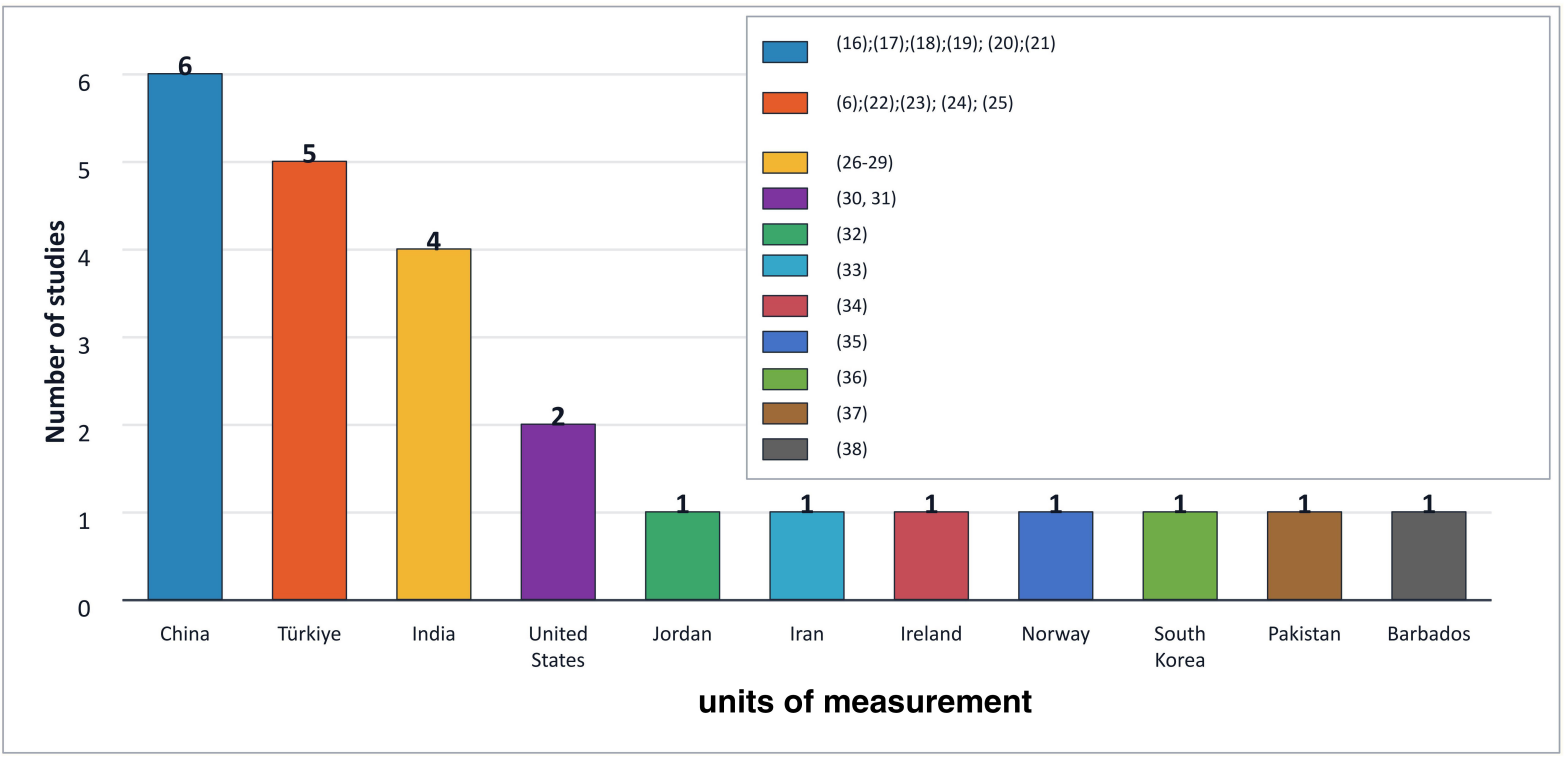
Geographical distribution of the included studies.

The included studies were conducted in 11 countries or regions according to the first author's primary affiliation. China was the most frequently represented country (*n* = 6) (16–21), followed by Türkiye (*n* = 5) (6, 22–25), India (*n* = 4) (26–29), and the United States (*n* = 2) (30, 31). One study each was conducted in Jordan (32), Iran (33), Ireland (34), Norway (35), South Korea (36), Pakistan (37), and Barbados (38). The details are shown in Figure 2, Geographical distribution of the included studies.

### 3.2 Topic categories and output types

General diabetes education, diabetes self-management education and support (DSMES), or primary-care diabetes questions were most frequent (*n* = 6), followed jointly by type 2 diabetes education or management and gestational diabetes mellitus (*n* = 4 each). The details are shown in Table 1.

**Table 1:** Topic categories and output types.

Topic category	Frequency, *n*	Studies
General diabetes education/diabetes self-management/primary care diabetes questions	6	(26); (18);(22); (35); (37);(21)
Type 2 diabetes-specific education or management	4	(34); (36); (25); (38)
Gestational diabetes mellitus	4	(17); (6); (33); (24)
Diabetic retinopathy	3	(16); (31); (29)
Diabetes medication education	2	(27); (28)
Discharged patients with diabetes	1	(19)
Diabetic kidney disease	1	(20)
Diabetic foot ulcer	1	(30)
Diabetes technology education	1	(23)
Mixed diabetes and endocrine queries	1	(32)
Output types	Frequency, *n*	Studies
Patient-facing answers to questions/FAQs	18	(26); (16); (17); (6); (33);(34); (30); (22); (35);(23); (31); (24); (32); (36);(37); (25); (29); (21)
Patient education materials/guides/brochures/education texts	5	(18); (19); (20); (27); (28)
Summarized majority responses from repeated generation	1	(38)

Regarding AI output types, most studies evaluated patient-facing answers to questions or FAQs (*n* = 18). Five studies evaluated patient education materials, guides, brochures, or educational texts. One study evaluated summarized majority responses generated from repeated AI outputs. The details are shown in Table 1.

### 3.3 Model characteristics

**Figure 3:**
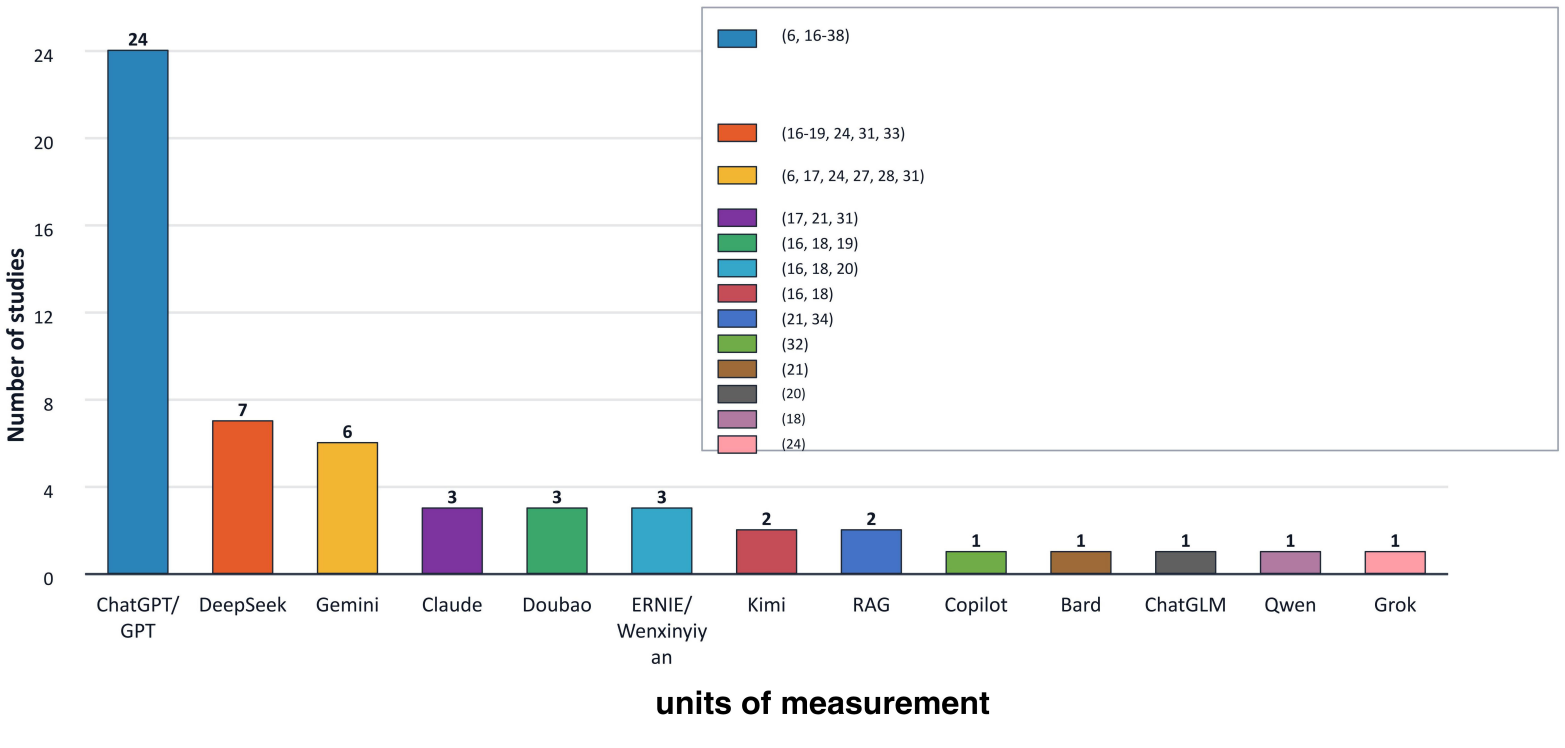
Model characteristics.

The AI models evaluated included ChatGPT or GPT-family models in all 24 studies. Other models or platforms included DeepSeek (*n* = 7) (16–19, 24, 31, 33), Gemini (*n* = 6) (6, 17, 24, 27, 28, 31), Claude (*n* = 3) (17, 21, 31), Doubao (*n* = 3) (16, 18, 19), ERNIE Bot or Wenxinyiyan (*n* = 3) (16, 18, 20), Kimi (*n* = 2) (16, 18), and retrieval-augmented generation systems or workflows (*n* = 2) (21, 34). Other models or platforms were reported in one study each, including Microsoft Copilot (32), Google Bard (21), ChatGLM (20), Qwen (18), and Grok (24). The details are shown in Figure 3, Model Characteristics.

### 3.4 Information quality evaluation indicators

A total of 11 indicators were mapped to the information quality framework. The intrinsic IQ dimension comprised accuracy (*n* = 13), believability (*n* = 9), and objectivity (*n* = 1). The contextual IQ dimension included completeness (*n* = 8), relevancy (*n* = 6), value-added (*n* = 4), and appropriate amount of data (*n* = 3). The representational IQ dimension encompassed ease of understanding (*n* = 14), interpretability (*n* = 2), and representational consistency (*n* = 3). Finally, the accessibility IQ dimension comprised accessibility (*n* = 1).

Six additional outcome indicators were identified: clinical safety (*n* = 5), actionability (*n* = 3), response efficiency (*n* = 1), personalization (*n* = 1), transparency (*n* = 1), and empathy (*n* = 1). Details are presented in Table 2.

**Table 2:** Information quality evaluation indicators.

Framework category	Consolidated indicator	Studies, *n*	Original terms and source studies
Intrinsic IQ	Accuracy	13	Accuracy: Abbasi et al. (32); Cheng et al. (20); Faraji et al. (33); Rohrich et al. (30); Senoymak et al. (22); D. Wang et al. (21); Cang et al. (16); Zha et al. (19); Skjervold et al. (35). content accuracy: Abuzar et al. (26). validity: Chung and Chang (36). comparative accuracy: Faisal et al. (37) false/misleading information: Gokbulut et al. (25)
Intrinsic IQ	Believability	9	Reliability: Karnan et al. (27); Saji et al. (28); Tekin et al. (23); Gokbulut et al. (25); X. Wang et al. (17); Yalcin et al. (24). trustworthiness: Abuzar et al. (26). source attribution/source matching: Kelly et al. (34). unreliable: Hernandez et al. (38)
Intrinsic IQ	Objectivity	1	Gender discrepancy/bias: Wu et al. (31)
Contextual IQ	Completeness	8	Completeness: Abbasi et al. (32); Cheng et al. (20); Faraji et al. (33); Subramanian et al. (29). comprehensiveness: Rohrich et al. (30); Faisal et al. (37); D. Wang et al. (21). adequacy: Senoymak et al. (22)
Contextual IQ	Relevancy	6	Relevance: Abbasi et al. (32); Abuzar et al. (26). appropriateness: Kelly et al. (34); Subramanian et al. (29). clinical appropriateness: Yalcin et al. (24). appropriate/inappropriate: Hernandez et al. (38)
Contextual IQ	Value-added	4	Utility: Tekin et al. (23); Chung and Chang (36). helpfulness: Skjervold et al. (35). effectiveness: Zha et al. (19)
Contextual IQ	Appropriate amount of data	3	Response length: Cang et al. (16). word count: Z. Wang et al. (18); Yalcin et al. (24). sentence count: Z. Wang et al. (18). syllable count: Z. Wang et al. (18)
Representational IQ	Ease of understanding	14	Readability: Abuzar et al. (26); Karnan et al. (27); Aypar Akbag (6); Saji et al. 2025 (28); Rohrich et al. (30); Tekin et al. (23); Z. Wang et al. (18); Wu et al. (31); Gokbulut et al. (25); X. Wang et al. (17); Yalcin et al. (24). understandability: Aypar Akbag (6); Z. Wang et al. (18); D. Wang et al. (21). Comprehensibility: Zha et al. (19); Yalcin et al. (24). Patient comprehensibility: Cheng et al. (20)
Representational IQ	Interpretability	2	Clarity: Abuzar et al. (26). logical consistency: Cang et al. (16). Coherence: Cang et al. (16)
Representational IQ	Representational consistency	3	Reproducibility: Senoymak et al. (22); Gokbulut et al. (25). Semantic similarity: Kelly et al. (34)
Accessibility IQ	Accessibility	1	Content accessibility: Cang et al. (16)
Additional outcome	Consolidated indicator	Studies, *n*	Original terms and source studies
Additional outcome	Clinical safety	5	Safety: Cang et al. (16); Zha et al. (19); Cheng et al. (20); Chung and Chang (36). danger: Rohrich et al. (30)
Additional outcome	Actionability	3	Actionability: Aypar Akbag (6); Z. Wang et al. (18); Zha et al. (19)
Additional outcome	Response efficiency	1	Response time: Cang et al. (16)
Additional outcome	Personalization	1	Personalization: Zha et al. (19)
Additional outcome	Transparency	1	JAMA benchmark criteria/benchmark adherence-transparency: X. Wang et al. (17)
Additional outcome	Empathy	1	Empathy: Skjervold et al. (35)

### 3.5 Framework-mapped information quality outcomes

Due to variations among evaluators and assessment methods, we describe the results only for the three most frequently used information quality indicators. Details are provided in Supplementary Document 7.

Among the 14 studies evaluating ease of understanding, the most common measures were the Flesch-Kincaid Grade Level and Flesch Reading Ease, each used in 9 studies (64.3%), followed by the Gunning Fog Index in 7 studies (50.0%), SMOG in 5 studies (35.7%), Coleman-Liau Index in 4 studies (28.6%), PEMAT-P understandability or comprehensibility assessment in 3 studies (21.4%). Automated Readability Index and patient-rated five-point understandability or comprehensibility scores each in 2 studies (14.3%). The Linsear Write Formula, Ateşman readability formula, and an average grade level based on multiple readability indices were each used in one study (7.1%).

Across most studies, AI-generated diabetes-related information required reading levels above those generally recommended for patient education, although some context-specific materials demonstrated more favorable readability and understandability. Abuzar reported a mean Flesch-Kincaid Grade Level of 16.87, indicating approximately college-level reading difficulty (26). Rohrich et al. reported a mean reading grade level of 11.9, corresponding to approximately 12th-grade readability (30). Tekin et al. reported a mean Flesch-Kincaid Grade Level of 10.5, corresponding to approximately 10th- to 11th-grade readability (23). Wu et al. similarly found that all evaluated models generated responses at high-school to college reading levels (31). Studies using PEMAT-P or patient-rated assessments suggested that understandability or comprehensibility could be acceptable in some contexts, but this did not necessarily translate into sufficient actionability. For example, Zha et al. (19) reported high PEMAT-P comprehensibility pass rates but lower actionability pass rates. Tekin et al. (23) showed that a simplification prompt improved readability substantially. Cheng et al. (20) assessed patient comprehensibility using a patient-rated five-point Likert scale rather than a formal readability formula and noted that the absence of a recognized Chinese readability assessment tool limited objective readability evaluation. An exception was the gestational diabetes educational material evaluated by Aypar Akbag (6), which achieved an Ateşman readability score of 77.8 and high PEMAT-P understandability (91.36%) and actionability (89.70%) scores. However, its Gunning Fog Index remained relatively high (16.25), indicating that conclusions regarding readability varied according to the assessment method used.

Among the 13 studies evaluating accuracy, most used study-specific Likert-type ratings, or categorical classification rules (11/13, 84.6%). One study used the CLEAR tool, and one used guideline-based expert review against ADA standards to identify false or misleading information.

Several studies reported high accuracy ratings, including maximum or near-maximum content-accuracy ratings, no inaccurate responses (19, 22, 25, 26). However, accuracy was not uniform across studies. Some responses that were judged accurate were still incomplete or omitted key information, indicating that factual correctness did not necessarily ensure sufficient comprehensiveness for patient education (30, 36). Comparative studies also showed that accuracy varied by model, with some newer or retrieval-enhanced models performing better than comparators (16, 19, 21, 33). One multilingual study found that Urdu responses were usually less accurate than English responses (37).

Among the 9 studies evaluating believability, 6 used DISCERN-related tools, including DISCERN, modified DISCERN, or mDISCERN. The remaining studies used study-specific rating.

The findings suggested generally favorable but not uniformly high believability. Model-comparison studies showed that reliability differed across AI systems. ChatGPT-5 achieved the highest DISCERN score in X. Wang et al. (17), whereas Grok and Gemini showed higher mDISCERN scores than ChatGPT (24). Studies using study-specific ratings also reported favorable findings, with Abuzar et al. (26) showing high trustworthiness scores and Hernandez et al. (38) reporting no unreliable responses. Kelly et al. (34) added a different dimension of believability by assessing whether RAG-generated responses could be traced to reference documents. Most cited responses were source-matched, although some relied on general model knowledge.

### 3.6 Additional outcomes

Clinical safety was assessed most frequently (*n* = 5), followed by actionability (*n* = 3), response efficiency (*n* = 1), personalization (*n* = 1), transparency (*n* = 1), and empathy (*n* = 1). Clinical safety was evaluated using heterogeneous, study-specific expert-rating scales (16, 19, 20, 30, 36). Although the studies generally reported low perceived risk or relatively high safety ratings, the findings could not be directly compared because of differences in definitions, scales, and thresholds. Actionability was assessed more consistently using the PEMAT-P (6, 18, 19). However, the results varied considerably, with reported scores ranging from a median of 20.0% to a mean of 89.7%, partly reflecting differences in topics and evaluated materials. Response efficiency was assessed in one study based on the time required to generate a complete response. ChatGPT-5 demonstrated the shortest mean response time (15.92 ± 4.48 s), with significant differences observed across the evaluated models (*P* < 0.001) (16). The single studies examining personalization (19) and empathy (35) reported generally favorable ratings, although between-model differences were small or nonsignificant. Transparency was limited: using the JAMA benchmark criteria, nearly all evaluated outputs received scores of zero (17). Details are provided in Supplementary Document 8.

### 3.7 Methodological structure

The details of methodological structure are presented in Table 3, Methodological structure.

**Table 3:** Methodological structure.

References	Prompt language	Session structure	AI input types	Prompting strategy	Evaluator types	Blinding	Inter-rater agreement	Tool types
Abuzar et al. (26)	English	S1	A; C	Zero-shot/direct prompting	H	B3	NR	T2; T3
Cang et al. (16)	Chinese	S1	A; C	Zero-shot/direct prompting	H	B1	QIA	T2; T4
X. Wang et al. (17)	Chinese	S1	A; G	Zero-shot/direct prompting	H	B3	QIA	T1; T3; T4
Z. Wang et al. (18)	English	S1	A	Zero-shot/direct prompting	H	B1	QIA	T1; T3
Zha et al. (19)	Chinese	S1	D	Role prompting	H	B2	QIA	T1; T2; T4
Aypar Akbag (6)	Turkish	S1	A; C	Zero-shot/direct prompting	H	B3	QIA	T1; T3; T4
Cheng et al. (20)	Chinese	NR	B; D	Role prompting	H + P	B2	NR	T2; T4
Faraji et al. (33)	English	NR	A; C	Zero-shot/direct prompting	H	B1	QA	T2; T4
Karnan et al. (27)	English	NR	B	Zero-shot/direct prompting	NR	B3	NR	T1; T3
Kelly et al. (34)	English	NR	A; E	Role prompting	H	B3	QA	T2; T4
Rohrich et al. (30)	English	S2	A; F	Zero-shot/direct prompting	H	B3	NR	T2; T3
Saji et al. (28)	English	NR	B	Zero-shot/direct prompting	NR	B3	NR	T1; T3
Senoymak et al. (22)	English	NR	A	Zero-shot/direct prompting	H	B3	QIA	T2; T4
Skjervold et al. (35)	Norwegian	NR	A	Zero-shot/direct prompting	H + P	B2	NR	T2
Tekin et al. (23)	English and Turkish	S1	A; F	Zero-shot/direct prompting	H	B3	NR	T1; T2; T3
Wu et al. (31)	English	NR	F	Zero-shot/direct prompting	NR	B1	NR	T2; T3; T4
Yalcin et al. (24)	English	S2	A; C; F	Zero-shot/direct prompting	H	B1	QIA	T1; T3; T4
Abbasi et al. (32)	English and Arabic	S1	A	Zero-shot/direct prompting	H	B3	QIA	T1
Chung and Chang (36)	English	NR	A	Zero-shot/direct prompting	H	B3	QA	T2; T4
Faisal et al. (37)	Urdu and English	NR	A	Zero-shot/direct prompting	H	B3	NR	T2
Gokbulut et al. (25)	Not clearly reported	S1	A; C; F	Zero-shot/direct prompting	H	B2	QIA	T1; T2; T3; T4
Subramanian et al. (29)	English	NR	A	Zero-shot/direct prompting	H	B3	QIA	T2; T4
D. Wang et al. (21)	English	S1	A; E	Zero-shot/direct prompting	H + P	B1	QA	T2; T4
Hernandez et al. (38)	English	NR	A	Zero-shot/direct prompting	H	B3	QA	T2; T4

### 3.8 Prompt characteristics

Prompt characteristics included prompt language, session structure, AI input types, prompting strategy, and retrieval architecture.

Prompt language was reported as English in 14 studies, Chinese in 4 studies, Turkish in 1 study, Norwegian in 1 study, Urdu and English in 1 study, English and Arabic in 1 study, English and Turkish in 1 study, and was unclear in 1 study. These non-English/non-Chinese prompts were evaluated within English-language publications.

Session structure was operationally defined as how prompts or questions were entered into the AI model across conversational contexts, including whether each prompt was entered in a new or reset session and whether all prompts were entered within the same continuous conversation. Based on this definition, 10 studies used independent or new sessions for each prompt, 2 studies used a continuous single session, and 12 studies did not report the session structure.

AI input types were heterogeneous. Patient or public questions and FAQs were most common (*n* = 19). Guideline- or authoritative-source-derived questions (*n* = 6), clinical scenarios (*n* = 5), PEM-generation prompts (*n* = 3), uploaded documents or records (*n* = 2), retrieval-augmented reference corpora (*n* = 2), and MCQ/exam items (*n* = 1) were also used.

“Prompting strategies” refers to the way in which prompts were structured to elicit AI-generated responses or educational materials. In this review, “zero-shot/direct prompting” referred to directly asking a question or instructing the model to generate content without providing examples; “few-shot prompting” referred to prompts that provided one or more examples or demonstrations for the model to follow; and “role prompting” referred to prompts that explicitly asked the model to act as a physician, expert, health educator, or similar role. Zero-shot/direct prompting was the most frequently used strategy (*n* = 21), followed by role prompting (*n* = 3). No study clearly reported the use of few-shot prompting.

Two studies explicitly implemented RAG workflows (21, 34), whereas the remaining 22 studies did not report controlled retrieval. D. Wang et al. directly compared identical questions with and without RAG and reported a mean 12% increase in accuracy and a 0.44-point increase in comprehensiveness (21). Kelly found that 94% of document-grounded responses were fully appropriate, compared with 50% of responses generated from general model knowledge, although no matched non-RAG comparison was performed (34).

### 3.9 Evaluator types and methodological reporting

Evaluator types were classified into three categories. Eighteen studies used healthcare professionals, three studies used both healthcare professionals and patients/public end users, and three studies did not clearly report evaluator type or used reviewers with unspecified qualifications.

Evaluator blinding was classified into three categories: full source/model blinding, partial or limited blinding, and not reported or no source/model blinding. Six studies clearly reported full source/model blinding, four reported partial or limited blinding, and 14 did not report blinding or did not implement source/model blinding.

Inter-rater agreement was classified into three categories: quantitative agreement reported, qualitative agreement process reported, and not reported or not applicable. Quantitative inter-rater agreement statistics were reported in 10 studies (41.7%). Five studies (20.8%) described qualitative agreement processes, such as consensus discussion or adjudication, without reporting quantitative agreement statistics. The remaining 9 studies (37.5%) did not report inter-rater agreement or stated that it was not calculated or not applicable.

### 3.10 Tool types

Across the 24 included studies, study-specific or self-developed evaluation tools were most frequently used (17/24, 70.8%), followed by clinical/reference standard-based assessment (15/24, 62.5%), readability formulas (11/24, 45.8%), and established instruments or reporting benchmarks (10/24, 41.7%). This indicates that most studies relied on locally developed scoring rubrics or classification systems, while fewer used established assessment instruments such as DISCERN, modified DISCERN, PEMAT-P, EQIP, GQS, JAMA benchmarks, CLEAR tools.

## 4 Discussion

### 4.1 Evidence gaps in evaluating AI-generated diabetes patient education materials

This scoping review shows that research on AI-generated PEMs for diabetes has evolved from basic ChatGPT Q&A to the assessment of various models and languages. However, the evidence base remains predominantly focused on commonly studied and broadly applicable topics, such as general diabetes education, Type 2 diabetes management, and gestational diabetes. On the other hand, there is relatively less research on more complex, specialized areas such as diabetic nephropathy, diabetic foot care, and discharge education (19, 20, 30). This conclusion is consistent with the findings of Aydin et al. and AlSammarraie and Househ on research on large language models for patient education (8, 9). Diabetes education should be factually correct but also include measures of understandability and safety. Therefore, future research should move from the question “Can AI answer patients' questions?” to “Can AI safely, understandably, and practically support patient self-management of specific diabetes education tasks?” (3, 4).

Readability formulas are used to measure ease of understanding. This is in line with the long tradition of focusing on reading grade levels in the literature on patient education materials. However, readability formulas mainly reflect word length, sentence length, and text complexity. They are best used as preliminary screening measures, rather than as single indicators of patient understanding or behavioral change (19). This difference is particularly important for the present review. The studies reviewed were conducted in different languages, such as English, Chinese, Turkish, and Arabic. However, many of the classic readability formulas were not originally designed for non-English texts. Faisal noticed performance differences when the same model was applied to Urdu and English diabetes-related Q&A content, which shows that the language context can influence the quality of AI output and assessment outcomes (37). Therefore, future research should use PEMAT-P, patient understanding and actionability assessment tools, and culturally appropriate multilingual tools (6, 18).

The heterogeneity is especially high for the accuracy evaluation. The majority of the studies use self-developed scoring systems or Likert scales, indicating the lack of standardized criteria for the evaluation of accuracy in this field. In studies, “accuracy” may mean factual accuracy, guideline adherence, congruence with expert consensus, completeness of the response, or absence of misleading information (16, 17, 22, 26). This variation explains why some studies demonstrate a high degree of accuracy in AI responses but also identify issues such as omission, incompleteness, or unclear boundaries of clinical applicability (30, 36). Regarding patient education for diabetes, factual accuracy is a fundamental requirement. Clinical accuracy should not be restricted to superficial fact checking or agreement with clinical guidelines. It should also evaluate the consistency of AI-generated content with pathophysiological mechanisms underlying diabetes. If it lacks risk warnings or specific advice as to what behavior to follow, then it may not be appropriate for direct delivery to patients. Inconsistency in evaluation tools can lead to evaluators' subjective judgment and measurement bias, making it harder to compare results from different studies. This highlights the need for standardized evaluation protocols and reporting checklists tailored to generative AI applications in healthcare (39). Future research should apply validated evaluation tools or adapt them appropriately. If new evaluation tools need to be developed, clear scoring criteria should be specified and rigorous psychometric validation should be performed.

The assessment of believability has been mostly based on tools such as DISCERN. DISCERN was not originally developed for generative AI but to assess the reliability of written consumer health information (40). Beyond the typical concerns about the quality of sources, the trustworthiness issues with generative AI include fabricated citations, untraceable evidence, expressions of overconfidence, delays in model updates, and inconsistent responses to the same query (41–43). Recent work suggests that patients are increasingly turning to general-purpose large language models for medical advice, which may contain online misinformation or outdated guidelines (44). The issue is that patients may be unable to verify the accuracy of the information, and they could harm themselves by making health decisions based on it. Therefore, future assessments of believability should be expanded to include authenticity, traceability, and content alignment, and they should be verified by patients or clinicians.

The measurement of these six additional outcomes could be further refined. Clinical safety was generally determined using study-specific expert ratings, with limited differentiation among error types, clinically important omissions, and levels of potential harm. Although the PEMAT-P provided a more consistent approach to actionability (45), it primarily indicates whether readers can identify recommended actions rather than whether those actions are accurate, safe, feasible, or appropriate for individual patients. Response efficiency may reflect network conditions, server workload, and platform infrastructure in addition to model performance. Personalization and empathy were largely based on single-item subjective ratings, whereas the JAMA criteria emphasized basic disclosure practices without fully addressing source reliability or claim-level traceability. These findings should therefore be interpreted within the scope of the respective measures. Future research may benefit from validated multidimensional instruments with clearly defined constructs and standardized scoring criteria to improve comparability across studies.

### 4.2 Methodological heterogeneity and limited patient involvement in AI-generated diabetes education research

From a methodological perspective, this review reveals significant variations in session structure, prompt language, prompting strategies, and the backgrounds of evaluators.

Half of the studies did not report the session structure, which makes it difficult to evaluate the influence of cumulative context on model output and thus affects the interpretability and reproducibility of the findings. Prompt language also affects output quality. The generated content would be affected by the training corpora of models, the wording of medical terminology, and the cultural backgrounds of patients, especially in multilingual and non-English studies (31, 37). In health education for diabetes, AI-generated content with inaccurate terminology, inadequate risk warnings, or poor cultural appropriateness in non-English contexts may impede patients' adherence to self-management advice.

For prompting strategies, zero-shot (or direct) prompting was adopted in 21 studies, role prompting was adopted in three studies, and no studies used few-shot prompting. This suggests that the current work is mainly evaluating the model performance on the direct-query setting. In a study of frequently asked questions for total knee arthroplasty patients, Chen et al. compared zero-shot prompting with role prompting and found that role prompting could improve the accuracy, comprehensiveness, and acceptability of responses from some models (46). This shows that role prompting strategies can affect the quality of AI-generated materials.

Beyond prompt strategies, system-level grounding represents another methodological dimension affecting output quality. Compared with standard zero-shot or role-prompted outputs, RAG-based systems can generate responses based on retrieved external knowledge sources, such as clinical guidelines, curated educational materials, or domain-specific databases (47). This approach may improve factual accuracy, believability, and traceability. RAG may reduce the risk of hallucinated or unsupported statements. However, only two of the 24 included studies explicitly implemented RAG workflows. Wang et al. provided a direct within-study comparison and reported improvements in accuracy and comprehensiveness after RAG implementation (21), whereas Kelly et al. evaluated source grounding without a matched non-RAG comparator (34). The limited number of RAG-based studies and the heterogeneity of evaluation methods precluded a definitive conclusion that RAG-based outputs were consistently superior to standard prompting approaches. Future studies should compare RAG-based systems with zero-shot and role-prompted outputs using standardized metrics.

Only three of the studies included in this review involved patients or members of the public in the assessment process. This suggests that the current research is still largely based on expert judgment, study-specific scoring, or automated tools, and not on the actual user experience of end-users. This gap is particularly crucial for patients with diabetes. Patients need to understand and act on complex information about diet, exercise, and other factors to manage their diabetes themselves. Educational materials may be accurate or comprehensive, according to experts, but that doesn't mean patients will understand them or act appropriately. Previous research has identified a substantial discrepancy between the readability of educational resources and the reading abilities of the general population (48). Future research should therefore involve patients in the development of standardized, validated, multilingual, and patient-centered assessment tools.

### 4.3 An expanded information quality model for evaluating AI-generated diabetes education materials

Although Wang and Strong's information quality framework provided a useful theoretical structure for organizing traditional information quality indicators, it was not originally developed for generative AI-generated health information. Therefore, it does not fully capture several concerns that are particularly relevant to AI-generated patient education. Several important indicators, including clinical safety, actionability, response efficiency, personalization, transparency, and empathy, could not be directly mapped to the original framework. This suggests that the evaluation of AI-generated diabetes education materials requires an expanded conceptual model that integrates both traditional information quality dimensions and additional dimensions.

**Figure 4:**
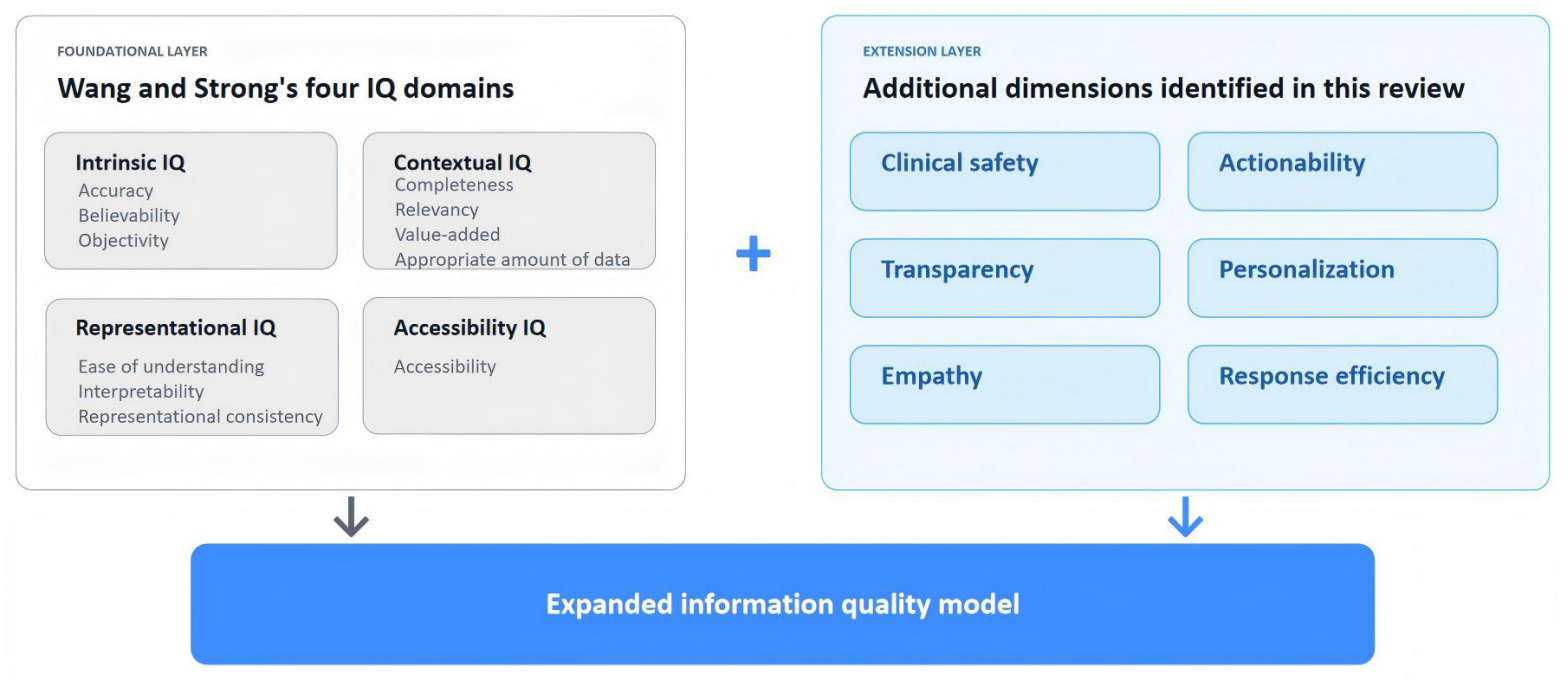
Expanded information quality model for evaluating AI-generated diabetes education materials.

To better reflect these findings, we propose an expanded conceptual model (Figure 4) that retains the four domains of Wang and Strong's framework while incorporating additional dimensions identified in this review, including clinical safety, actionability, transparency, personalization, empathy, and response efficiency. This extended model represents a preliminary conceptual framework proposed based on the findings of this review and has not yet undergone external validation or psychometric testing.

## 5 Limitations

This review has several limitations. Although eight databases and citation searching were used, the search was limited to Chinese- and English-language publications, and relevant studies in other languages may have been missed. The restriction to Chinese- and English-language publications may have resulted in the omission of studies evaluating models in other linguistic contexts. In keeping with the purpose of a scoping review, we did not formally assess the methodological quality or risk of bias of the included studies, so the findings should be interpreted with caution. Another limitation is the rapid pace at which generative AI systems change. Results based on a specific model version may no longer apply after updates to training data or system functions. In addition, the included studies differed in session structure, prompt language, prompting strategies, and the backgrounds of evaluators, which makes direct comparison difficult. Only a small number of studies involved patients or members of the public, so the perspectives of people living with diabetes, particularly those with limited health literacy, remain underrepresented. Most studies assessed the quality of generated text rather than clinical outcomes, such as changes in patient knowledge or self-management behavior.

Our search did not cover major computer science and informatics databases. Although the review focused on clinical evaluations rather than technical AI development, relevant studies published in computer science venues may have been missed. This may have favored clinically oriented measures, such as readability formulas, DISCERN, PEMAT-P, and clinician-rated accuracy, while underrepresenting model-specific evaluations of hallucination, robustness, and reproducibility.

## 6 Conclusions

This review identified a rapidly expanding yet methodologically heterogeneous literature on AI-generated diabetes education. Clinical safety, actionability, personalization, transparency, empathy and response efficiency have been less explored in current studies, which have mainly focused on accuracy, believability and ease of understanding. Although AI-generated materials demonstrated favorable performance in some settings, differences in models, prompts, languages, evaluators, and scoring criteria prevent broad conclusions about their suitability for direct patient use. Future studies should adopt transparent and reproducible reporting practices, involve people living with diabetes, and develop validated multilingual tools that assess not only whether information is accurate and understandable, but also whether it can safely and practically support diabetes self-management.

## Data Availability

The original contributions presented in the study are included in the article/Supplementary material, further inquiries can be directed to the corresponding author/s.
