# Supplementary material for: Assessing the information quality of AI-generated patient educational materials for diabetes: a scoping review
Source: Front Public Health. 2026 Aug 5;14:1902424. doi: 10.3389/fpubh.2026.1902424 (PMC13485973; doi:10.3389/fpubh.2026.1902424)
Supplement: Supplementary file 1 [file Supplementary_File_1.docx]

**Supplementary 1. Database Search Strategies**

Search date limit: inception to May 1, 2026

# PubMed(1085)

Use publication date filter: `Publication dates: to 2026/05/01`, or add `AND ("1800/01/01"[Date - Publication] : "2026/05/01"[Date - Publication])`.

("Diabetes Mellitus"[Mesh] OR diabet*[Title/Abstract] OR prediabet*[Title/Abstract] OR "type 1 diabetes"[Title/Abstract] OR "type 2 diabetes"[Title/Abstract] OR "gestational diabetes"[Title/Abstract] OR "diabetic retinopathy"[Title/Abstract] OR "diabetic foot"[Title/Abstract] OR "diabetic foot ulcer*"[Title/Abstract] OR "diabetic neuropath*"[Title/Abstract] OR "diabetic nephropath*"[Title/Abstract])

AND

("artificial intelligence"[MeSH] OR "AI"[Title/Abstract] OR "generative AI"[Title/Abstract] OR "large language model"[Title/Abstract] OR LLM[Title/Abstract] OR "language model"[Title/Abstract] OR chatbot[Title/Abstract] OR "chat bot"[Title/Abstract] OR chatbots[Title/Abstract] OR "conversational agent"[Title/Abstract] OR "conversational agents"[Title/Abstract] OR "dialogue system"[Title/Abstract] OR "dialogue systems"[Title/Abstract] OR "natural language processing"[MeSH] OR ChatGPT[Title/Abstract] OR "GPT-3.5"[Title/Abstract] OR "GPT-4"[Title/Abstract] OR "GPT-4o"[Title/Abstract] OR "GPT-4 Turbo"[Title/Abstract] OR "GPT-3.5-turbo"[Title/Abstract] OR "GPT-o1"[Title/Abstract] OR Gemini[Title/Abstract] OR Bard[Title/Abstract] OR "Google Bard"[Title/Abstract] OR Claude[Title/Abstract] OR Copilot[Title/Abstract] OR Perplexity[Title/Abstract] OR ERNIE[Title/Abstract] OR ChatGLM[Title/Abstract] OR "generative pre-trained transformer"[Title/Abstract] OR OpenAI[Title/Abstract] OR "foundation model"[Title/Abstract] OR "foundation models"[Title/Abstract])

AND

("patient education as topic"[MeSH] OR "patient education"[Title/Abstract] OR "health education"[MeSH] OR "health education"[Title/Abstract] OR "patient information"[Title/Abstract] OR "patient material"[Title/Abstract] OR "patient materials"[Title/Abstract] OR "educational material"[Title/Abstract] OR "educational materials"[Title/Abstract] OR "health information"[Title/Abstract] OR "patient question"[Title/Abstract] OR "patient questions"[Title/Abstract] OR "patient query"[Title/Abstract] OR "patient queries"[Title/Abstract] OR FAQ[Title/Abstract] OR "frequently asked question"[Title/Abstract] OR "frequently asked questions"[Title/Abstract] OR "patient response"[Title/Abstract] OR "patient responses"[Title/Abstract] OR counselling[Title/Abstract] OR counseling[Title/Abstract] OR "health literacy"[MeSH] OR "health literacy"[Title/Abstract] OR "self-management"[Title/Abstract] OR "self care"[MeSH] OR "self-care"[Title/Abstract] OR "discharge education"[Title/Abstract] OR "patient discharge"[MeSH] OR "consumer health information"[MeSH] OR "health communication"[MeSH] OR "question answering"[Title/Abstract] OR "Q&A"[Title/Abstract] OR "health messaging"[Title/Abstract] OR "patient education handout"[Title/Abstract] OR "health information quality"[Title/Abstract] OR "patient-directed"[Title/Abstract] OR "patient facing"[Title/Abstract] OR "patient-facing"[Title/Abstract] OR "decision support"[Title/Abstract] OR "self-care support"[Title/Abstract])

# Web of Science Core Collection(897)

Use topic search. Set Timespan from all years to May 1, 2026 if the interface supports date filtering; otherwise export records and filter by publication date.

TS=(diabet* OR prediabet* OR "type 1 diabetes" OR "type 2 diabetes" OR "gestational diabetes" OR "diabetic retinopathy" OR "diabetic foot" OR "diabetic foot ulcer*" OR "diabetic neuropath*" OR "diabetic nephropath*")

AND

TS=("artificial intelligence" OR AI OR "generative AI" OR "large language model" OR LLM OR "language model" OR chatbot OR "chat bot" OR chatbots OR "conversational agent" OR "conversational agents" OR "dialogue system" OR "dialogue systems" OR "natural language processing" OR ChatGPT OR "GPT-3.5" OR "GPT-4" OR "GPT-4o" OR "GPT-4 Turbo" OR "GPT-3.5-turbo" OR "GPT-o1" OR Gemini OR Bard OR "Google Bard" OR Claude OR Copilot OR Perplexity OR ERNIE OR ChatGLM OR "generative pre-trained transformer" OR OpenAI OR "foundation model" OR "foundation models")

AND

TS=("patient education" OR "health education" OR "patient information" OR "patient material" OR "patient materials" OR "educational material" OR "educational materials" OR "health information" OR "patient question" OR "patient questions" OR "patient query" OR "patient queries" OR FAQ OR "frequently asked question" OR "frequently asked questions" OR "patient response" OR "patient responses" OR counselling OR counseling OR "health literacy" OR "self-management" OR "self care" OR "self-care" OR "discharge education" OR "patient discharge" OR "consumer health information" OR "health communication" OR "question answering" OR "Q&A" OR "health messaging" OR "patient education handout" OR "health information quality" OR "patient-directed" OR "patient facing" OR "patient-facing" OR "decision support" OR "self-care support")

# Embase(1636)

('diabetes mellitus'/exp OR diabet*:ti,ab,kw OR prediabet*:ti,ab,kw OR 'type 1 diabetes':ti,ab,kw OR 'type 2 diabetes':ti,ab,kw OR 'gestational diabetes':ti,ab,kw OR 'diabetic retinopathy':ti,ab,kw OR 'diabetic foot':ti,ab,kw OR 'diabetic foot ulcer*':ti,ab,kw OR 'diabetic neuropath*':ti,ab,kw OR 'diabetic nephropath*':ti,ab,kw)

AND

('artificial intelligence'/exp OR 'natural language processing'/exp OR AI:ti,ab,kw OR 'generative AI':ti,ab,kw OR 'large language model':ti,ab,kw OR LLM:ti,ab,kw OR 'language model':ti,ab,kw OR chatbot:ti,ab,kw OR 'chat bot':ti,ab,kw OR chatbots:ti,ab,kw OR 'conversational agent':ti,ab,kw OR 'conversational agents':ti,ab,kw OR 'dialogue system':ti,ab,kw OR 'dialogue systems':ti,ab,kw OR ChatGPT:ti,ab,kw OR 'GPT-3.5':ti,ab,kw OR 'GPT-4':ti,ab,kw OR 'GPT-4o':ti,ab,kw OR 'GPT-4 Turbo':ti,ab,kw OR 'GPT-3.5-turbo':ti,ab,kw OR 'GPT-o1':ti,ab,kw OR Gemini:ti,ab,kw OR Bard:ti,ab,kw OR 'Google Bard':ti,ab,kw OR Claude:ti,ab,kw OR Copilot:ti,ab,kw OR Perplexity:ti,ab,kw OR ERNIE:ti,ab,kw OR ChatGLM:ti,ab,kw OR 'generative pre-trained transformer':ti,ab,kw OR OpenAI:ti,ab,kw OR 'foundation model':ti,ab,kw OR 'foundation models':ti,ab,kw)

AND

('patient education'/exp OR 'health education'/exp OR 'health literacy'/exp OR 'self care'/exp OR 'patient education':ti,ab,kw OR 'health education':ti,ab,kw OR 'patient information':ti,ab,kw OR 'patient material':ti,ab,kw OR 'patient materials':ti,ab,kw OR 'educational material':ti,ab,kw OR 'educational materials':ti,ab,kw OR 'health information':ti,ab,kw OR 'patient question':ti,ab,kw OR 'patient questions':ti,ab,kw OR 'patient query':ti,ab,kw OR 'patient queries':ti,ab,kw OR FAQ:ti,ab,kw OR 'frequently asked question':ti,ab,kw OR 'frequently asked questions':ti,ab,kw OR 'patient response':ti,ab,kw OR 'patient responses':ti,ab,kw OR counselling:ti,ab,kw OR counseling:ti,ab,kw OR 'self-management':ti,ab,kw OR 'self care':ti,ab,kw OR 'self-care':ti,ab,kw OR 'discharge education':ti,ab,kw OR 'patient discharge':ti,ab,kw OR 'consumer health information':ti,ab,kw OR 'health communication':ti,ab,kw OR 'question answering':ti,ab,kw OR 'Q&A':ti,ab,kw OR 'health messaging':ti,ab,kw OR 'patient education handout':ti,ab,kw OR 'health information quality':ti,ab,kw OR 'patient-directed':ti,ab,kw OR 'patient facing':ti,ab,kw OR 'patient-facing':ti,ab,kw OR 'decision support':ti,ab,kw OR 'self-care support':ti,ab,kw)

# Scopus(2200)

Use date filter: `PUBDATETXT` or interface filter to publication date on or before May 1, 2026. If only year filter is available, limit to 2026 and manually remove records after May 1, 2026.

TITLE-ABS-KEY(diabet* OR prediabet* OR "type 1 diabetes" OR "type 2 diabetes" OR "gestational diabetes" OR "diabetic retinopathy" OR "diabetic foot" OR "diabetic foot ulcer*" OR "diabetic neuropath*" OR "diabetic nephropath*")

AND

TITLE-ABS-KEY("artificial intelligence" OR AI OR "generative AI" OR "large language model" OR LLM OR "language model" OR chatbot OR "chat bot" OR chatbots OR "conversational agent" OR "conversational agents" OR "dialogue system" OR "dialogue systems" OR "natural language processing" OR ChatGPT OR "GPT-3.5" OR "GPT-4" OR "GPT-4o" OR "GPT-4 Turbo" OR "GPT-3.5-turbo" OR "GPT-o1" OR Gemini OR Bard OR "Google Bard" OR Claude OR Copilot OR Perplexity OR ERNIE OR ChatGLM OR "generative pre-trained transformer" OR OpenAI OR "foundation model" OR "foundation models")

AND

TITLE-ABS-KEY("patient education" OR "health education" OR "patient information" OR "patient material" OR "patient materials" OR "educational material" OR "educational materials" OR "health information" OR "patient question" OR "patient questions" OR "patient query" OR "patient queries" OR FAQ OR "frequently asked question" OR "frequently asked questions" OR "patient response" OR "patient responses" OR counselling OR counseling OR "health literacy" OR "self-management" OR "self care" OR "self-care" OR "discharge education" OR "patient discharge" OR "consumer health information" OR "health communication" OR "question answering" OR "Q&A" OR "health messaging" OR "patient education handout" OR "health information quality" OR "patient-directed" OR "patient facing" OR "patient-facing" OR "decision support" OR "self-care support")

# Cochrane CENTRAL(168)

Use Cochrane CENTRAL advanced search. Search all text, title, abstract and keywords. Apply publication date to May 1, 2026 where available.

(diabet* OR prediabet* OR "type 1 diabetes" OR "type 2 diabetes" OR "gestational diabetes" OR "diabetic retinopathy" OR "diabetic foot" OR "diabetic foot ulcer*" OR "diabetic neuropath*" OR "diabetic nephropath*")

AND

("artificial intelligence" OR AI OR "generative AI" OR "large language model" OR LLM OR "language model" OR chatbot OR "chat bot" OR chatbots OR "conversational agent" OR "conversational agents" OR "dialogue system" OR "dialogue systems" OR "natural language processing" OR ChatGPT OR "GPT-3.5" OR "GPT-4" OR "GPT-4o" OR "GPT-4 Turbo" OR "GPT-3.5-turbo" OR "GPT-o1" OR Gemini OR Bard OR "Google Bard" OR Claude OR Copilot OR Perplexity OR ERNIE OR ChatGLM OR "generative pre-trained transformer" OR OpenAI OR "foundation model" OR "foundation models")

AND

("patient education" OR "health education" OR "patient information" OR "patient material" OR "patient materials" OR "educational material" OR "educational materials" OR "health information" OR "patient question" OR "patient questions" OR "patient query" OR "patient queries" OR FAQ OR "frequently asked question" OR "frequently asked questions" OR "patient response" OR "patient responses" OR counselling OR counseling OR "health literacy" OR "self-management" OR "self care" OR "self-care" OR "discharge education" OR "patient discharge" OR "consumer health information" OR "health communication" OR "question answering" OR "Q&A" OR "health messaging" OR "patient education handout" OR "health information quality" OR "patient-directed" OR "patient facing" OR "patient-facing" OR "decision support" OR "self-care support")

# CNKI(24)

主题=(糖尿病 + 糖尿病前期 + 2型糖尿病 + 1型糖尿病 + 妊娠期糖尿病 + 糖尿病视网膜病 + 糖尿病足溃疡 +糖尿病足 + 糖尿病神经病变 + 糖尿病肾病)

AND

主题=(人工智能 + 生成式人工智能 + 大语言模型 + 大型语言模型 + 语言模型 + 自然语言处理 + 聊天机器人 + 对话机器人 + 对话系统 + 会话代理 + 智能问答 + AI + ChatGPT + "GPT-3.5" + "GPT-4" + "GPT-4o" + "GPT-4 Turbo" + "GPT-3.5-turbo" + "GPT-o1" + Gemini + Bard + "Google Bard" + Claude + Copilot + Perplexity + ERNIE + 文心一言)

AND

主题=(患者教育 + 病人教育 + 健康教育 + 患者信息 + 病人信息 + 患者材料 + 教育材料 + 健康信息 + 患者问题 + 病人问题 + 患者提问 + 患者咨询 + 常见问题 + 问答 + 咨询 + 健康素养 + 自我管理 + 自我护理 + 自我照护 + 出院教育 + 出院指导 + 患者出院 + 消费者健康信息 + 健康传播 + 健康沟通 + 健康信息质量 + 患者导向 + 面向患者 + 患者面对 + 决策支持 + 自我管理支持)

# WanFang Data(18)

主题:(糖尿病 OR 糖尿病前期 OR 1型糖尿病 OR 2型糖尿病 OR 妊娠期糖尿病 OR 糖尿病视网膜病变 OR 糖尿病足 OR 糖尿病足溃疡 OR 糖尿病神经病变 OR 糖尿病肾病)

AND

主题:(人工智能 OR 生成式人工智能 OR 大语言模型 OR 大型语言模型 OR 语言模型 OR 自然语言处理 OR 聊天机器人 OR 对话机器人 OR 对话系统 OR 会话代理 OR 智能问答 OR AI OR ChatGPT OR "GPT-3.5" OR "GPT-4" OR "GPT-4o" OR "GPT-4 Turbo" OR "GPT-3.5-turbo" OR "GPT-o1" OR Gemini OR Bard OR "Google Bard" OR Claude OR Copilot OR Perplexity OR ERNIE OR 文心一言)

AND

主题:(患者教育 OR 病人教育 OR 健康教育 OR 患者信息 OR 病人信息 OR 患者材料 OR 教育材料 OR 健康信息 OR 患者问题 OR 病人问题 OR 患者提问 OR 患者咨询 OR 常见问题 OR 问答 OR 咨询 OR 健康素养 OR 自我管理 OR 自我护理 OR 自我照护 OR 出院教育 OR 出院指导 OR 患者出院 OR 消费者健康信息 OR 健康传播 OR 健康沟通 OR 健康信息质量 OR 患者导向 OR 面向患者 OR 患者面对 OR 决策支持 OR 自我管理支持)

# SinoMed(21)

Use SinoMed advanced search. If subject headings are available, combine Chinese subject headings with title/abstract/free-text terms. Limit publication date to May 1, 2026.

主题:(糖尿病 OR 糖尿病前期 OR 1型糖尿病 OR 2型糖尿病 OR 妊娠期糖尿病 OR 糖尿病视网膜病变 OR 糖尿病足 OR 糖尿病足溃疡 OR 糖尿病神经病变 OR 糖尿病肾病)

AND

主题:(人工智能 OR 生成式人工智能 OR 大语言模型 OR 大型语言模型 OR 语言模型 OR 自然语言处理 OR 聊天机器人 OR 对话机器人 OR 对话系统 OR 会话代理 OR 智能问答 OR AI OR ChatGPT OR "GPT-3.5" OR "GPT-4" OR "GPT-4o" OR "GPT-4 Turbo" OR "GPT-3.5-turbo" OR "GPT-o1" OR Gemini OR Bard OR "Google Bard" OR Claude OR Copilot OR Perplexity OR ERNIE OR 文心一言)

AND

主题:(患者教育 OR 病人教育 OR 健康教育 OR 患者信息 OR 病人信息 OR 患者材料 OR 教育材料 OR 健康信息 OR 患者问题 OR 病人问题 OR 患者提问 OR 患者咨询 OR 常见问题 OR 问答 OR 咨询 OR 健康素养 OR 自我管理 OR 自我护理 OR 自我照护 OR 出院教育 OR 出院指导 OR 患者出院 OR 消费者健康信息 OR 健康传播 OR 健康沟通 OR 健康信息质量 OR 患者导向 OR 面向患者 OR 患者面对 OR 决策支持 OR 自我管理支持)
