# Supplementary material for: Assessing the information quality of AI-generated patient educational materials for diabetes: a scoping review
Source: Front Public Health. 2026 Aug 5;14:1902424. doi: 10.3389/fpubh.2026.1902424 (PMC13485973; doi:10.3389/fpubh.2026.1902424)
Supplement: Supplementary file 2 [file Supplementary_File_2.docx]

**Supplementary 2. Abbreviations and Full Terms**

| **Abbreviation** | **Full term** |
| --- | --- |
| AAO | American Academy of Ophthalmology |
| ACM | Association for Computing Machinery |
| ACSM | American College of Sports Medicine |
| ADA | American Diabetes Association |
| AI | artificial intelligence |
| ARI | Automated Readability Index |
| AUC | area under the receiver operating characteristic curve |
| CENTRAL | Cochrane Central Register of Controlled Trials |
| CHART | Chatbot Assessment Reporting Tool |
| CI | confidence interval |
| CLEAR | CLEAR tool; the included article reported that it assessed completeness, accuracy, and relevance/appropriateness, but did not provide a full expansion of the acronym |
| CLI | Coleman-Liau Index |
| CNKI | China National Knowledge Infrastructure |
| CVI | Content Validity Index |
| DAI | Diabetic Association of India |
| DFU | diabetic foot ulcer |
| DKD | diabetic kidney disease |
| DM | diabetes mellitus |
| DOI | digital object identifier |
| DR | diabetic retinopathy |
| DSMES | Diabetes Self-Management Education and Support |
| EHR | electronic health record |
| EQIP | Ensuring Quality Information for Patients |
| EQIP-36 | 36-item Ensuring Quality Information for Patients instrument |
| FAQ | frequently asked question |
| FAQs | frequently asked questions |
| FKGL | Flesch-Kincaid Grade Level |
| FRE | Flesch Reading Ease |
| FRES | Flesch Reading Ease Score |
| GBD | Global Burden of Disease |
| GDM | gestational diabetes mellitus |
| GFI | Gunning Fog Index |
| GFOG | Gunning Fog Index |
| GLP-1 | glucagon-like peptide-1 |
| GPT | Generative Pre-trained Transformer |
| GQS | Global Quality Scale |
| ICC | intraclass correlation coefficient |
| IDF | International Diabetes Federation |
| IEEE | Institute of Electrical and Electronics Engineers |
| IQ | information quality |
| JAMA | Journal of the American Medical Association |
| IQR | interquartile range |
| JBI | Joanna Briggs Institute |
| LLM | large language model |
| MCQ | multiple-choice question |
| METRICS | METRICS checklist for generative AI health-care studies |
| mDISCERN | modified DISCERN instrument |
| NCD | non-communicable disease |
| NLP | natural language processing |
| OSF | Open Science Framework |
| PCC | Population-Concept-Context |
| PEM | patient education material |
| PEMs | patient education materials |
| PEMAT | Patient Education Materials Assessment Tool |
| PEMAT-P | Patient Education Materials Assessment Tool for Printable Materials |
| PPP | Preferred Practice Pattern |
| PRISMA | Preferred Reporting Items for Systematic Reviews and Meta-Analyses |
| PRISMA-ScR | Preferred Reporting Items for Systematic Reviews and Meta-Analyses Extension for Scoping Reviews |
| RAG | retrieval-augmented generation |
| RISE | Retrieval-augmented Information System for Enhancement |
| SMOG | Simple Measure of Gobbledygook |
| STROBE | Strengthening the Reporting of Observational Studies in Epidemiology |
| T1DM | type 1 diabetes mellitus |
| T2DM | type 2 diabetes mellitus |
| TTR | type-token ratio |
