# Supplementary material for: Assessing the information quality of AI-generated patient educational materials for diabetes: a scoping review
Source: Front Public Health. 2026 Aug 5;14:1902424. doi: 10.3389/fpubh.2026.1902424 (PMC13485973; doi:10.3389/fpubh.2026.1902424)
Supplement: Supplementary file 3 [file Supplementary_File_3.docx]

**Supplementary 3. Extracted variables, operational definitions, and coding**

| Variable category | Extracted variables | Operational definition |
| --- | --- | --- |
| Study characteristics | Author, publication year, country or region | Country or region was defined according to the first author’s primary affiliation. |
| Diabetes-related topic | Main disease/topic category | Studies were categorized by their main diabetes-related focus, such as general diabetes education, type 2 diabetes management, gestational diabetes mellitus, diabetic retinopathy, diabetes medication education, discharge education, diabetic kidney disease, diabetic foot ulcer, diabetes technology education, or mixed diabetes/endocrine queries. |
| AI output type | Patient-facing answers, FAQs, patient education materials, guides, brochures, educational texts, summarized repeated outputs | Output type was defined according to the final form of AI-generated content evaluated in each study. |
| AI model characteristics | AI model name and version | Reported AI models name and version were extracted. ChatGPT, GPT-3.5, GPT-4, GPT-4o and other GPT versions were grouped as GPT-family, while specific model names were retained. |
| Prompt language | Language used in prompts or input materials | Recorded as reported by the study, such as English, Chinese, Turkish, Norwegian, Urdu and English, English and Arabic, or not clearly reported. |
| Session structure | New or reset session, same continuous session, and not reported | Defined according to whether prompts were entered in new or reset sessions, within the same continuous conversation, or whether this information was not reported. |
| AI input types | Patient/public questions, FAQs, guideline-derived questions, clinical scenarios, PEM-generation prompts, uploaded documents or records,Retrieval-augmented reference corpora, MCQ/exam items | Input type was defined according to the source and format of materials entered into the AI model. Categories were not mutually exclusive. |
| Prompting strategy | Zero-shot/direct prompting, role prompting, few-shot prompting | Zero-shot/direct prompting referred to direct questioning or content generation without examples; role prompting referred to instructing the AI to act as a physician, expert, educator, or similar role; few-shot prompting referred to prompts containing examples. |
| Evaluator type | Evaluator types | Evaluators were classified as healthcare professionals, healthcare professionals plus patients/public end users, and Not clearly reported.  Healthcare professionals included physicians, specialists, nurses, diabetes educators, and personnel involved in clinical assessment/health communication |
| Evaluator blinding | Full source/model blinding  ,Partial/limited blinding, not reported | Full source/model blinding  The original text explicitly states that the evaluator is unaware of which model or source provided the response, and this applies to the study's primary manual evaluation process.  Partial/limited blinding  Blinding is applied only to a subset of evaluators, specific evaluation stages, or arbitrators; alternatively, materials may be anonymized or randomly numbered without explicit stipulation in the original text that all evaluators remain blind to the model or source.  not reported  The blinding method was not reported, or the original text indicates that source/model blinding was not performed. |
| Inter-rater agreement | ICC、Cohen’s kappa、weighted kappa、Kendall’s W、Cronbach’s alpha、Jaccard agreement、percent agreement, consensus/adjudication | Inter-rater agreement was defined as whether and how consistency between independent evaluators was assessed or handled during the evaluation of AI-generated outputs. It was coded as QIA when quantitative agreement statistics, such as ICC, Cohen’s kappa, weighted kappa, Kendall’s W, Jaccard coefficient, or percentage agreement, were reported; QA when disagreements were handled through discussion, consensus, adjudication, or consultation with an additional reviewer without reporting a quantitative agreement statistic; and NR when inter-rater agreement was not reported, not calculated, or not applicable. |
| Tool types | Clinical/reference standards, study-specific scoring, readability formulas, DISCERN, GQS, PEMAT-P, EQIP, CLEAR, JAMA benchmarks | Tool types were coded only when the original study reported a clearly identifiable assessment instrument, formula, scoring rubric, classification system, or reference-based evaluation standard. General expert judgement alone was not coded as a tool type unless it was linked to explicit scoring criteria or a defined reference standard. |
| Information-quality indicators | Ease of understanding, accuracy, believability, completeness, relevancy, value-added, representational consistency, appropriate amount of data, interpretability, objectivity, accessibility | Indicators were first retained using the original terms reported by the authors and then mapped to Wang and Strong’s information-quality framework. Similar concepts were consolidated. |
| Separate category | Clinical safety, actionability, response efficiency, personalization, transparency, empathy | Indicators that were evaluated but could not be primarily mapped to Wang and Strong’s framework were retained as additional outcomes. |

*Coding and Corresponding Definitions*

Session structure

S1-new or reset session; S2-same continuous conversation; NR-Not clearly reported

AI input types

A-Patient/public questions or FAQs; B-PEM-generation prompts; C-Guideline- or authoritative-source-derived questions; D-Uploaded documents/records; E-Retrieval-augmented reference corpora; F-Clinical scenarios; G-MCQ/exam items

Evaluator types

H-Healthcare professionals; H+P -Healthcare professionals plus patients/public end users; NR-Not clearly reported

Blinding

B1-Full source/model blinding; B2-Partial/limited blinding; B3-Not reported or no source/model blinding

Inter-rater agreement

QIA-Quantitative inter-rater agreement reported; QA-Qualitative agreement process reported; NR-Not reported / not applicable

Tool types

T1-Established assessment instruments or reporting benchmarks; T2-Study-specific/self-developed evaluation tools; T3-Readability formulas; T4-Clinical/reference standard-based assessment
