# Supplementary material for: Assessing the information quality of AI-generated patient educational materials for diabetes: a scoping review
Source: Front Public Health. 2026 Aug 5;14:1902424. doi: 10.3389/fpubh.2026.1902424 (PMC13485973; doi:10.3389/fpubh.2026.1902424)
Supplement: Supplementary file 4 [file Supplementary_File_4.docx]

| Domains | Dimensions | Theoretical meaning |
| --- | --- | --- |
| Intrinsic Information Quality | Accuracy, Believability, Objectivity, Reputation | Data have quality in their own right. This category concerns whether data are intrinsically good. |
| Contextual Information Quality | Relevancy, Value-added, Timeliness, Completeness, Appropriate amount of data | Data quality must be considered within the context of the task at hand. This category concerns whether data are contextually appropriate for the task. |
| Representational Information Quality | Interpretability, Ease of understanding (Understandability), Representational consistency, Conciseness | This category concerns whether data are clearly represented, interpretable, easy to understand, concisely represented, and consistently represented. |
| Accessibility Information Quality | Accessibility, Access security | High-quality data should be accessible to the data consumer. |

**Supplementary 4. Theoretical framework details**
