# Supplementary material for: Assessing the information quality of AI-generated patient educational materials for diabetes: a scoping review
Source: Front Public Health. 2026 Aug 5;14:1902424. doi: 10.3389/fpubh.2026.1902424 (PMC13485973; doi:10.3389/fpubh.2026.1902424)
Supplement: Supplementary file 5 [file Supplementary_File_5.docx]

**Supplementary Document 5: Coding Matrix**

| **Framework category** | **Consolidated indicator** | **Operational definition / coding rule** |
| --- | --- | --- |
| Intrinsic IQ | Accuracy | Coded when the study assessed factual, medical, scientific, or guideline-level correctness, including absence of false or misleading information. |
| Intrinsic IQ | Believability | Coded when the study assessed credibility, reliability, trustworthiness, or whether responses were supported by identifiable sources. |
| Intrinsic IQ | Objectivity | Coded when the study evaluated bias or differential representation across patient characteristics. |
| Contextual IQ | Completeness | Coded when the study assessed whether the response covered all necessary content or omitted key information. |
| Contextual IQ | Relevancy | Coded when the study assessed fit between the AI response and the user question, clinical scenario, or education context. |
| Contextual IQ | Value-added | Coded when the study assessed usefulness, utility, helpfulness, or perceived added educational value. |
| Contextual IQ | Appropriate amount of data | Coded when response length or amount of information was evaluated as information load or content quantity. |
| Representational IQ | Ease of understanding | Coded only when readability, understandability, or comprehensibility was assessed with a tool, formula, rating scale, or reported quantitative result. Descriptive statements without measurement were not coded. |
| Representational IQ | Interpretability | Coded when the study assessed clarity of expression, internal logic, or coherence within a response. |
| Representational IQ | Representational consistency | Coded when the study assessed consistency across repeated prompts, sessions, or semantically comparable outputs. |
| Accessibility IQ | Accessibility | Coded when the study assessed ease of access or patient-facing accessibility of the content. |
| Additional outcome | Clinical safety | Coded when the study assessed potential harm, unsafe advice, danger, or clinical safety risk. |
| Additional outcome | Actionability | Coded when the study assessed whether the material provided concrete, executable guidance for patients. |
| Additional outcome | Response efficiency | Coded only when the study measured AI model response generation time or latency. Participant evaluation time was not included. |
| Additional outcome | Personalization | Coded when the study assessed adaptation to patient-specific needs or contexts. |
| Additional outcome | Transparency | Coded when the study assessed compliance with transparency or benchmark-reporting criteria. |
| Additional outcome | Empathy | Coded when the study assessed perceived empathy, communication quality, or user preference. |
