# Supplementary material for: Assessing the information quality of AI-generated patient educational materials for diabetes: a scoping review
Source: Front Public Health. 2026 Aug 5;14:1902424. doi: 10.3389/fpubh.2026.1902424 (PMC13485973; doi:10.3389/fpubh.2026.1902424)
Supplement: Supplementary file 6 [file Supplementary_File_6.docx]

**Supplementary 6. General characteristics of the 24 included studies**

| **Study** | **Country** | **Topic** | **AI outputs evaluated** | **AI model/version** | **Evaluation indicators** |
| --- | --- | --- | --- | --- | --- |
| Abuzar et al. | India | General diabetes education | 10 ChatGPT-4o responses | ChatGPT-4o | Overall quality; content accuracy; clarity; relevance; trustworthiness; readability; response length. |
| Cang et al. | China | Diabetic retinopathy | 150 responses: 30 questions x 5 models | ChatGPT-5; DeepSeek-V3.1; Doubao; Wenxinyiyan 4.5 Turbo; Kimi | Accuracy; logical consistency; coherence; safety; content accessibility; response efficiency and structure. |
| X. Wang et al. | China | Gestational diabetes mellitus | MCQ task: 3,600 attempts: 200 MCQs x 6 models x 3 runs. Public education task: 60 responses: 15 questions x 4 models | ChatGPT-5; ChatGPT-4o; DeepSeek-V3.2; DeepSeek-R1; Gemini 2.5 Pro; Claude Sonnet 4.5 | Validity/accuracy; reliability; information quality; global quality; benchmark adherence/transparency; readability; MCQ accuracy. |
| Z. Wang et al. | China | Diabetes self-management education | 200 education texts: 20 prompts x 10 platforms | ChatGPT 4o; ChatGPT 5.1-Thinking; ChatGPT 5.2-Thinking; DeepSeek-R1; Doubao; ERNIE Bot 5.0 Preview; Kimi K2; Qwen3; Qwen3-Max; Qwen3-Max-Thinking-Preview | Overall quality; understandability; actionability; information quality; readability; text characteristics. |
| Zha et al. | China | Discharged patients with diabetes | 30 health education materials: 10 records x 3 models; expert-rating records = 150 | ChatGPT-4o; Doubao 1.5; DeepSeek R1 | Comprehensibility/understandability; actionability; accuracy; personalization; effectiveness; safety. |
| Aypar Akbağ | Turkey | Gestational diabetes mellitus | 64 AI-generated responses/materials: 32 questions x 2 platforms | ChatGPT; Gemini; exact versions not reported | Understandability; actionability; readability; content and quality. |
| Cheng et al. | China | Early diabetic kidney disease | 7 AI-generated PEMs; 8 PEMs expert-evaluated when including 1 physician-generated PEM; 2 top PEMs evaluated by 50 patients | ERNIE Bot 4.0; GPT-4o; ChatGLM4; ChatGPT-o1 | Accuracy; completeness; safety; patient comprehensibility. |
| Faraji et al. | Iran | Gestational diabetes mellitus | 72 AI responses: 24 questions x 3 chatbots; 8 resident responses per question used as comparator | GPT-3.5; GPT-4o; DeepSeek V3 0324 | Accuracy; completeness; clinical reliability discussed. |
| Karnan et al. | India | GLP-1 receptor agonist medication education | 6 education guides: 3 medications x 2 models | ChatGPT-3.5; Google Gemini | Readability; similarity/overlap; reliability; word count. |
| Kelly et al. | Ireland | Type 2 diabetes health literacy | Main evaluation: 44 one-off responses plus 16 simulated consultation responses. Repeatability analysis: each of the 44 questions was queried 20 times | Custom RAG chatbot using OpenAI’s GPT-4o mini | Appropriateness; source attribution/source matching; response grounding. |
| Rohrich et al. | United States | Diabetic foot ulcers | 11 ChatGPT-3.5 responses | ChatGPT-3.5 | Accuracy; comprehensiveness; danger/safety; readability. |
| Saji et al. | India | Common antidiabetic medication education | 12 education guides: 6 drugs x 2 models | ChatGPT version 4.0;  Gemini version 1.5 | Readability; reliability; similarity; text characteristics. |
| Şenoymak et al. | Turkey | Diabetes-related primary care questions | 64 ChatGPT responses | ChatGPT GPT-3.5, Nov 12, 2023 version | Accuracy; adequacy; reproducibility. |
| Skjervold et al. | Norway | Diabetes patient education and health guidance | 113 GPT-4o responses; 113 healthcare professional responses used as comparator | GPT-4o version 2024-08-06 | Perceived knowledge; helpfulness; empathy; response time; participant preference/relative perceived quality. |
| Tekin et al. | Turkey | Diabetes technology education | 40 language-specific responses generated; main quantitative scoring reported for 20 English responses; 5 responses re-evaluated after simplification prompting | ChatGPT-4o | Reliability; quality; utility; readability; language differences. |
| Wu et al. | United States | Diabetic retinopathy/gender bias scenario | 8 responses: 1 scenario x 2 sex variants x 4 models | ChatGPT-o1; DeepSeek-v3; Gemini 2.0 Flash; Claude 3.7 Sonnet; ChatGPT-4.5 used for gender-bias comparison | Readability; clinical terminology frequency; healthcare recommendations; gender discrepancy/bias. |
| Yalcın et al. | Turkey | Gestational diabetes mellitus | 100 responses: 25 questions x 4 models | ChatGPT-4o; DeepSeek R-1; Gemini 2.5 Pro; Grok-3.0 | Reliability; content quality; overall usefulness; global quality; readability; lexical diversity; scientific accuracy; clinical appropriateness; comprehensibility. |
| Abbasi et al. | Jordan | mixed diabetes/endocrine queries | 80 total responses: 20 queries x 2 languages x 2 models; diabetes subset = 60 responses | ChatGPT-4o; Microsoft Copilot | Completeness; accuracy; relevance; overall CLEAR score. |
| Chung and Chang | South Korea | Type 2 diabetes exercise education | 14 ChatGPT responses | ChatGPT V.4.0 | Validity; safety; utility; readability. |
| Faisal et al. | Pakistan | Diabetes knowledge, lifestyle, and prevention | 106 responses: 53 Urdu responses plus 53 English responses | ChatGPT Model 3.5, March 2024 version | Comprehensiveness; comparative accuracy; cross-language consistency. |
| Gokbulut et al. | Turkey | Type 2 diabetes pharmacological management | 48 generated responses across two sessions; main DISCERN/readability analysis reported for 24 responses | ChatGPT-4 online platform, April 25, 2023 version | Reliability; treatment information quality; overall quality; readability; false/misleading information; consistency. |
| Subramanian et al. | India | Diabetic retinopathy | 20 ChatGPT-4 responses | ChatGPT-4 | Appropriateness; completeness. |
| D. Wang et al. | China | General diabetes education | 258 responses: 43 questions x 3 models x 2 conditions | GPT-4; Claude 2; Google Bard; RISE-enhanced versions; OpenAI Text-Embedding-ADA-002 for embeddings | Accuracy; comprehensiveness; understandability. |
| Hernandez et al. | Barbados | Type 2 diabetes and related complications | 210 raw responses generated; final evaluation used 70 summarized majority responses | ChatGPT version 3.5, August 2023 large language model | Appropriateness/reliability classification; reproducibility/consistency. |
