# Supplementary material for: Assessing the information quality of AI-generated patient educational materials for diabetes: a scoping review
Source: Front Public Health. 2026 Aug 5;14:1902424. doi: 10.3389/fpubh.2026.1902424 (PMC13485973; doi:10.3389/fpubh.2026.1902424)
Supplement: Supplementary file 7 [file Supplementary_File_7.docx]

**Supplementary Document 7: Reported outcomes for ease of understanding, accuracy, and believability**

| **Indicator** | **Study** | **Tool / method** | **Reported result** | **Narrative interpretation** |
| --- | --- | --- | --- | --- |
| Ease of understanding | Abuzar et al. 2026 | FRES, FKGL, SMOG, Gunning Fog | FRES 38.19 ± 8.42; FKGL 16.87 ± 2.31; SMOG 17.24 ± 1.98; Gunning Fog 19.63 ± 2.54 | The generated responses showed low reading ease and high grade-level readability scores, indicating that the English-language outputs were difficult for general patients to read. |
|  | Karnan et al. 2025 | Grade level, ease score | Grade level: ChatGPT 10.47 ± 0.86, Gemini 9.93 ± 1.10; ease score: ChatGPT 36.63 ± 2.24, Gemini 47.53 ± 5.13 | Gemini generated more readable patient education brochures than ChatGPT, whereas ChatGPT produced slightly more detailed content; readability differences were not statistically significant. |
|  | Aypar Akbağ 2025 | Ateşman, Gunning Fog, PEMAT-P | Ateşman: ChatGPT 66.8, Gemini 69.4, education material 77.8; Gunning Fog: ChatGPT 18.57, Gemini 17.54, education material 16.25; PEMAT-P understandability mean 91.36% | The findings were favorable for understandability and actionability, whereas readability conclusions varied across formulas |
|  | Saji et al. 2025 | Grade level, ease score | Grade level: ChatGPT 9.65 ± 0.65, Gemini 9.85 ± 0.67; ease score: ChatGPT 40.53 ± 3.92, Gemini 41.83 ± 3.95 | No meaningful difference in readability was observed between ChatGPT and Gemini-generated patient education brochures. |
|  | Rohrich et al. 2025 | Average reading grade level | Mean reading grade level 11.9 ± 1.8 | ChatGPT-generated responses were written at an average grade level of 11.9, indicating that the responses were above the usual recommended reading level for patient-facing materials. |
|  | Tekin et al. 2025 | FKGL, GFI, FRE | FKGL 10.5 ± 2.26; GFI 11.1 ± 3.03; FRE 37.8 ± 13.5. After simplification prompt: FKGL decreased from 10.4 to 4.9; GFI from 11.0 to 7.2; FRE increased from 37.1 to 79.0 | Readability was limited in the original ChatGPT-4o responses and required relatively high literacy; a simplification prompt substantially improved readability without changing the content. |
|  | Z. Wang et al. 2026 | PEMAT-P, ARI, FRES, GFOG, FKGL, CLI, SMOG, Linsear Write | PEMAT-P understandability 76.90 (66.70, 84.60); ARI 15.59; FRES 24.00; GFOG 15.40; FKGL 14.12; CLI 15.80; SMOG 12.30; LW 15.52 | Across platforms, generated diabetes education texts showed challenges in readability and actionability; readability varied significantly by platform, and the materials often remained difficult for patient education use. |
|  | Wu et al. 2025 | FRES, FKGL | FRES ranged 31.7-44.5; FKGL ranged 10.5-14.3 across evaluated model/sex-prompt outputs | All evaluated LLMs produced text at approximately tenth-grade to college reading levels; the authors concluded that improvements in readability remained necessary before patient use. |
|  | Gokbulut et al. 2024 | FRE, FKGL, GFI, CLI, SMOG | FRE 35.1 ± 9.9; FKGL 12.9 ± 2.0; GFI 16.3 ± 2.2; CLI 13.8 ± 1.1; SMOG 12.1 ± 1.6; reading level classified as difficult or very difficult in most responses | ChatGPT-4 responses were classified mostly as difficult or very difficult to read, despite generally acceptable reliability. |
|  | X. Wang et al. 2026 | ARI, CL, FKGL, FRES, GFI, SMOG | ChatGPT-5 showed the lowest difficulty among compared models: FKGL 7.43 ± 2.42, FRES 62.47 ± 13.51; other models generally had higher reading difficulty, e.g., Claude FKGL 13.11 ± 3.54, Gemini FKGL 13.04 ± 1.96 | All models produced text above the recommended sixth-grade reading level; ChatGPT-5 had the most favorable readability profile but still did not fully meet guideline targets. |
|  | Yalcin et al. 2025 | FRES, FKGL, SMOG, GFI, CLI | FKGL: ChatGPT 15.86 ± 3.83, DeepSeek 11.04 ± 1.65, Gemini 12.03 ± 1.89, Grok 17.06 ± 3.52; FRES all below 60 | All models scored below the Flesch Reading Ease threshold recommended for lay audiences, indicating that the responses were not sufficiently readable for general patients. |
|  | D. Wang et al. 2024 | Patient 1-5 understandability score | GPT-4 improved from 4.32 ± 0.61 to 4.64 ± 0.51 after RISE; Claude 4.01 ± 0.73 to 4.07 ± 0.74; Bard 3.96 ± 0.86 to 4.16 ± 0.82 | The RISE framework improved patient-rated understandability across the evaluated models, although the magnitude of improvement varied by model. |
|  | Zha et al. 2026 | PEMAT-P comprehensibility | Total score 0.80 (0.76, 0.85); pass rate 130/150 (86.7%); ChatGPT-4o 86.0%, Doubao 82.0%, DeepSeek R1 92.0% | Comprehensibility pass rates were generally high, with DeepSeek R1 performing best; however, actionability remained lower than comprehensibility. |
|  | Cheng et al. 2025 | 5-point Likert scale | 60% (30/50) of patients felt ERNIE Bot 4.0 performed as well as or better than physician PEM; preference varied by disease duration and proteinuria subgroup | Patient evaluation suggested that some AI-generated materials were considered understandable or preferable by patients, but the authors noted that the lack of a recognized Chinese readability assessment tool limited objective readability evaluation. |
| Accuracy | Abbasi et al. 2024 | CLEAR accuracy score | Accuracy scores across tools/categories: 4.35 ± 0.35; 4.02 ± 0.54; 4.08 ± 0.67; 3.58 ± 0.60 | AI-generated responses generally showed moderate-to-high accuracy, but accuracy varied across the evaluated tools or response categories. |
|  | Cheng et al. 2025 | 6-point Likert scale | Expert evaluation covered accuracy, completeness, and safety; extracted text reports ERNIE Bot 4.0 non-guideline PEM and physician PEM among the top-performing materials | Expert evaluation suggested that some AI-generated patient education materials performed well in accuracy-related assessment, but accuracy was evaluated together with completeness and safety rather than as a fully isolated outcome. |
|  | Faraji et al. 2025 | Study-specific 5-point score | Accuracy: residents 3.64; GPT-3.5 4.67; GPT-4o 4.69; DeepSeek V3 4.81; AI models scored significantly higher than residents | AI-generated diabetes education content was rated more accurate than resident-generated content, with DeepSeek V3 showing the highest accuracy among the evaluated AI models. |
|  | Rohrich et al. 2025 | 10-point physician rating | Mean accuracy 8.7 ± 0.3; however, 3/11 responses contained incorrect information and 10/11 omitted key information | The average accuracy rating was high, but some responses still contained incorrect information and most omitted key information, indicating that high accuracy scores did not guarantee completeness. |
|  | Şenoymak et al. 2025 | 3-point accuracy scale | Overall median accuracy 3.0 (IQR 3.0-3.0); no question received an inaccurate rating | ChatGPT responses were rated as highly accurate overall, and no response was classified as inaccurate. |
|  | D. Wang et al. 2024 | Clinician accuracy score and accuracy rate | Accuracy score: GPT-4 8.72 to 8.91; Claude 8.09 to 8.65; Bard 8.37 to 8.86. Accuracy rate improved from 91% to 98% for GPT-4, 72% to 91% for Claude, and 86% to 95% for Bard after RISE | Accuracy improved after applying the RISE retrieval-enhancement strategy across GPT-4, Claude, and Bard, suggesting that source-supported prompting can strengthen factual correctness. |
|  | Cang et al. 2026 | Study-specific 1-5 Likert rating | Accuracy median: ChatGPT-5 5.00; DeepSeek-V3.1 4.00; Doubao 4.00; Wenxinyiyan 4.00; Kimi 3.50; between-model difference significant | Accuracy differed significantly across models, with ChatGPT-5 receiving the highest accuracy rating and Kimi receiving the lowest among the evaluated models. |
|  | Zha et al. 2026 | Study-specific AI-based 5-point Likert-type evaluation tool | Accuracy pass rate: ChatGPT-4o 50/50 (100%); Doubao 49/50 (98%); DeepSeek R1 50/50 (100%) | ChatGPT-4o, Doubao, and DeepSeek R1 all achieved high accuracy pass rates, with ChatGPT-4o and DeepSeek R1 reaching full pass rates under the study’s predefined threshold. |
|  | Abuzar et al. 2026 | Study-specific 4-point content accuracy rating | Content accuracy 4.0 ± 0.0; median 4.0 | The generated content received the maximum content-accuracy rating, indicating consistently high accuracy under the study’s scoring system. |
|  | Chung and Chang 2024 | Validity 1-4 scale | 10/14 responses scored 4 for complete accuracy; 4/14 scored 3 for accurate but incomplete | Most responses were judged completely accurate, while the remaining responses were accurate but incomplete, indicating good factual validity with residual incompleteness. |
|  | Faisal et al. 2024 | Study-specific categorical expert assessment | Urdu response more accurate than English: 0/53; Urdu response less accurate than English: 49/53 (92.5%) | Urdu responses were usually less accurate than English responses, suggesting that language context may affect the accuracy of AI-generated diabetes information. |
|  | Skjervold et al. 2025 | 5-point Likert scale | Significant differences favored GPT-4o for perceived knowledge (χ²(4)=17.66, p=0.0014, Cramér’s V=0.099). | GPT-4o responses received slightly higher participant-perceived ratings for knowledge than responses from healthcare professionals, particularly among participants with lower educational attainment. |
|  | Gokbulut et al. 2024 | Guideline-based expert assessment against ADA 2023 standards | No false or misleading information was found | Expert assessment against ADA 2023 standards found no false or misleading information in ChatGPT-4 responses. |
| Believability | Karnan et al. 2025 | Modified DISCERN score | ChatGPT 4.00 ± 0.00; Gemini 3.67 ± 0.58. p = 0.4226. | Both ChatGPT and Gemini produced highly reliable patient education brochures, with no statistically significant difference in reliability between the two tools. |
|  | Saji et al. 2025 | Modified DISCERN score | ChatGPT 3.50 ± 0.55; Gemini 3.50 ± 0.55. p = 1.000. | ChatGPT and Gemini showed comparable moderate reliability, with no difference between models. |
|  | Tekin et al. 2025 | mDISCERN | Mean mDISCERN 28.4 ± 1.6; all 20 responses classified as fair reliability | ChatGPT-4o responses showed fair reliability overall; none reached the good reliability category. |
|  | Gokbulut et al. 2024 | DISCERN | DISCERN score 60.2 ± 1.9; reliability classification: good 21/24 (87.5%), excellent 3/24 (12.5%) | ChatGPT-4 responses were generally reliable, with most responses rated good and a smaller proportion rated excellent. |
|  | X. Wang et al. 2026 | DISCERN | ChatGPT-5 42.53 ± 7.20; Claude 30.67 ± 7.37; DeepSeek-V3.2 34.80 ± 6.10; Gemini 32.67 ± 8.30 | Reliability differed significantly across models, with ChatGPT-5 achieving the highest DISCERN score. |
|  | Yalcin et al. 2025 | mDISCERN | ChatGPT 25.40 ± 4.18; DeepSeek 27.23 ± 4.55; Gemini 28.49 ± 4.36; Grok 29.01 ± 6.01; all within moderate reliability category | All four models showed moderate reliability; Grok and Gemini scored highest, while ChatGPT scored lowest. |
|  | Abuzar et al. 2026 | Study-specific 4-point trustworthiness rating | Trustworthiness 3.9 ± 0.1; median 3.9 | ChatGPT-4o responses were rated highly trustworthy, although the narrow score range suggested a possible ceiling effect. |
|  | Kelly et al. 2025 | Four-category judgment | 32/44 (73%) responses correctly cited reference documents; 12/44 (27%) were general knowledge; no cited topic was absent from source documents | The RAG chatbot usually provided traceable source attribution, although some answers relied on general knowledge rather than reference documents. |
|  | Hernandez et al. 2023 | Study-specific physician classification | 69/70 responses were appropriate; 1/70 was inappropriate; no separate unreliable count was reported in the extracted table | Most ChatGPT responses were judged appropriate and none were classified as unreliable, although this was a categorical physician judgment rather than a standardized reliability instrument. |
