# Supplementary material for: Assessing the information quality of AI-generated patient educational materials for diabetes: a scoping review
Source: Front Public Health. 2026 Aug 5;14:1902424. doi: 10.3389/fpubh.2026.1902424 (PMC13485973; doi:10.3389/fpubh.2026.1902424)
Supplement: Supplementary file 8 [file Supplementary_File_8.docx]

**Supplementary Document 8 Reported outcomes for clinical safety, actionability, response efficiency, personalization, transparency and empathy**

| Additional outcome indicator | Studies, n (%) | Evaluation tools and methods | Quantitative findings | Measurement limitations |
| --- | --- | --- | --- | --- |
| Clinical Safety | 5 (20.8%) | All five studies used study-specific expert ratings. Cang et al. used a 5-point scale and assessed safety according to the presence of necessary referral advice; Zha et al. used a single-item 5-point scale; Cheng et al. used a 3-point scale; Rohrich et al. used a 10-point potential-danger scale; and Chung and Chang used a four-level safety scale. | Cang et al. reported median safety scores of 4 across the models, although between-model differences were significant (P=.004). In Zha et al., 78%–92% of responses received scores of ≥4 (P<.01). Rohrich et al. reported a mean danger score of 2.2±0.6, while Chung and Chang classified all 14 responses as presenting no danger.  Cheng et al. assessed safety using a three-point expert-rating scale. | No study used a validated, dedicated clinical safety instrument. Safety was variously operationalized as providing referral advice, presenting potential danger, or being unlikely to cause harm. These approaches did not systematically assess error type, harm severity, omissions, contraindications, or unsafe self-management behaviors. |
| Actionability | 3 (12.5%) | All three studies used the Patient Education Materials Assessment Tool for Printable Materials (PEMAT-P). Items were generally scored as 0 or 1 and converted to a percentage score; Zha et al. defined scores >70% as meeting the criterion. | Z. Wang et al. reported a median actionability score of 20.0% (IQR 0–40), with no difference between platforms (P=.313) but significant differences between topics (P<.001). Zha et al. reported median scores of approximately 0.80 and pass rates of 60%–64%. Aypar Akbağ reported a mean score of 89.7%. | PEMAT-P evaluates whether readers can identify actions from the material, but not whether those actions are clinically correct, safe, feasible, personalized, or implemented in practice. Aypar Akbağ evaluated a combined educational booklet, preventing attribution to an individual model. |
| Response efficiency | 1 (4.2%) | Cang et al. measured the time required for the models to generate complete responses. | Cang et al. measured the time from question submission to completion of the initial response. Mean response times differed significantly across models: ChatGPT-5, 15.92±4.48 seconds; Doubao, 27.07±2.91 seconds; Kimi, 34.41±2.37 seconds; DeepSeek-V3.1, 38.20±2.96 seconds; and Wenxinyiyan, 41.89±5.09 seconds (P<.001). | ChatGPT-5 produced responses fastest under the study conditions. However, the measured time represented user-perceived end-to-end latency and may have been influenced by network conditions, server load, and platform infrastructure; therefore, it should not be interpreted solely as intrinsic model efficiency. |
| Personalization | 1 (4.2%) | Zha et al. used a single-item, expert-rated 5-point scale, with scores ≥4 classified as good. | The proportions of responses rated ≥4 were 78% for ChatGPT-4o, 84% for Doubao, and 92% for DeepSeek, with no statistically significant difference (P=.14). | No validated personalization instrument was used. The assessment did not explicitly examine whether recommendations accounted for comorbidities, medications, health literacy, preferences, or individual circumstances. |
| Transparency | 1 (4.2%) | X. Wang et al. used the four JAMA benchmark criteria: authorship, attribution, currency, and disclosure. Each criterion was scored as absent (0) or present (1), producing a total score of 0–4. | ChatGPT-5 achieved a mean score of 0.07±0.26, whereas the other models scored 0. Although the difference was statistically significant (P=.0464), its absolute magnitude was negligible. Inter-rater reliability was ICC=1.00. | The JAMA criteria assess basic reporting transparency rather than citation accuracy, source quality, claim-level traceability, retrieval disclosure, or model uncertainty. |
| Empathy | 1 (4.2%) | Skjervold et al. used a study-specific questionnaire in which participants rated knowledge, helpfulness, and empathy on 5-point scales. | AI responses received higher ratings in 46.7% of comparisons, healthcare-professional responses in 23.3%, and equal ratings in 30.0%. Empathy ratings differed significantly (P<.001), but the effect was small (Cramér’s V=.115). | The questionnaire primarily measured perceived communication quality and lacked formal psychometric validation. It did not assess shared decision-making, trust, patient comprehension, or information retention. |
